# Baseline [^18^F]GTP1 tau PET imaging is associated with subsequent cognitive decline in Alzheimer’s disease

**DOI:** 10.1186/s13195-021-00937-x

**Published:** 2021-12-01

**Authors:** Edmond Teng, Paul T. Manser, Sandra Sanabria Bohorquez, Kristin R. Wildsmith, Karen Pickthorn, Suzanne L. Baker, Michael Ward, Geoffrey A. Kerchner, Robby M. Weimer

**Affiliations:** 1 Early Clinical Development, Genentech, Inc., South San Francisco, CA USA; 2 Clinical Biostatistics, Genentech, Inc., South San Francisco, CA USA; 3 Clinical Imaging Group, Genentech, Inc., South San Francisco, CA USA; 4 Biomarker Development, Genentech, Inc., South San Francisco, CA USA; 5 Molecular Biophysics and Integrated Bioimaging, Lawrence Berkeley National Laboratory, Berkeley, CA USA; 6 Current Address: Alector, Inc., South San Francisco, CA USA; 7 Current Address: F. Hoffmann-La Roche AG, Basel, Switzerland; 8 Biomedical Imaging, Genentech, Inc., South San Francisco, CA USA

**Keywords:** Tau, PET, Cognition, Alzheimer’s disease, Prognosis

## Abstract

**Background:**

The role and implementation of tau PET imaging for predicting subsequent cognitive decline in Alzheimer’s disease (AD) remains uncertain. This study was designed to evaluate the relationship between baseline [^18^F]GTP1 tau PET and subsequent longitudinal change across multiple cognitive measures over 18 months.

**Methods:**

Our analyses incorporated data from 67 participants, including cognitively normal controls (*n* = 10) and β-amyloid (Aβ)-positive individuals ([^18^F] florbetapir Aβ PET) with prodromal (*n* = 26), mild (*n* = 16), or moderate (*n* = 15) AD. Baseline measurements included cortical volume (MRI), tau burden ([^18^F]GTP1 tau PET), and cognitive assessments [Mini-Mental State Examination (MMSE), Clinical Dementia Rating (CDR), 13-item version of the Alzheimer’s Disease Assessment Scale-Cognitive Subscale (ADAS-Cog13), and Repeatable Battery for the Assessment of Neuropsychological Status (RBANS)]. Cognitive assessments were repeated at 6-month intervals over an 18-month period. Associations between baseline [^18^F]GTP1 tau PET indices and longitudinal cognitive performance were assessed via univariate (Spearman correlations) and multivariate (linear mixed effects models) approaches. The utility of potential prognostic tau PET cut points was assessed with ROC curves.

**Results:**

Univariate analyses indicated that greater baseline [^18^F]GTP1 tau PET signal was associated with faster rates of subsequent decline on the MMSE, CDR, and ADAS-Cog13 across regions of interest (ROIs). In multivariate analyses adjusted for baseline age, cognitive performance, cortical volume, and Aβ PET SUVR, the prognostic performance of [^18^F]GTP1 SUVR was most robust in the whole cortical gray ROI. When AD participants were dichotomized into low versus high tau subgroups based on baseline [^18^F]GTP1 PET standardized uptake value ratios (SUVR) in the temporal (cutoff = 1.325) or whole cortical gray (cutoff = 1.245) ROIs, high tau subgroups demonstrated significantly more decline on the MMSE, CDR, and ADAS-Cog13.

**Conclusions:**

Our results suggest that [^18^F]GTP1 tau PET represents a prognostic biomarker in AD and are consistent with data from other tau PET tracers. Tau PET imaging may have utility for identifying AD patients at risk for more rapid cognitive decline and for stratification and/or enrichment of participant selection in AD clinical trials.

Trial registration

ClinicalTrials.gov NCT02640092. Registered on December 28, 2015

**Supplementary Information:**

The online version contains supplementary material available at 10.1186/s13195-021-00937-x.

## Background

Neurofibrillary tangles (NFTs) comprised of hyperphosphorylated tau protein are one of the defining neuropathological characteristics of Alzheimer’s disease (AD) [1]. Several tau PET tracers label NFTs, including [^18^F]flortaucipir [2], [^18^F]GTP1 [3], [^18^F]RO948 [4], [^18^F]MK-6240 [5], and [^18^F]PI-2620 [6]. Tau PET imaging, particularly with second-generation tracers that exhibit less off-target binding [4], may represent an informative biomarker for tau pathology in AD, as evidenced by its inclusion in the National Institute on Aging-Alzheimer’s Association (NIA-AA) Research Framework for biological diagnosis of AD [7] and FDA approval of [^18^F]flortaucipir for assessing NFT burden in suspected AD-associated cognitive impairment [8].

Beyond their diagnostic utility, biomarkers may also help predict prognosis [9]. Some studies have demonstrated that [^18^F]flortaucipir, [^18^F]MK-6240, and [^18^F]RO948 tau PET correlate with cognitive decline in participants with AD and/or cognitively normal controls (particularly those with positive β-amyloid [Aβ] biomarkers) [10–17]. However, inconsistencies between reports suggest that relationships between baseline tau PET and longitudinal cognitive change may depend upon disease stage, specific assessments, and particular PET regions of interest (ROIs). Likewise, tau PET may interact with other variables (e.g., baseline cognition, Aβ indices, and/or cerebral atrophy) to predict future cognitive decline [18, 19].

Prior studies of tau PET versus cognitive change have examined continuous associations [11, 12] or dichotomized participant cohorts into low (T-) versus high (T+) tau PET [15]. Tau PET cutoffs have the potential to be used in AD clinical trials for stratification or inclusion/exclusion criteria to enrich for participants more likely to experience a subsequent decline. Existing T-/T+ distinctions have focused on diagnostic thresholds determined from cognitively normal Aβ-negative participants [20]. However, thresholds optimized for diagnostic accuracy may differ from those optimized for the prediction of cognitive outcomes.

The primary aim of this paper was to determine the prognostic utility of [^18^F]GTP1, a second-generation tau PET tracer that has been and is being used in multiple completed and ongoing therapeutic trials in AD, for identifying individuals at risk for more rapid clinical progression. In particular, we sought to confirm that associations between cognitive change and NFT pathology measured by [^18^F]GTP1 are consistent with those previously reported with [^18^F]flortaucipir [11, 17], [^18^F]MK-6240 [15], and [^18^F]RO948 [17], which would allow for future comparisons between studies using different tau PET tracers. We analyzed the relationship between baseline [^18^F]GTP1 tau PET indices and longitudinal cognitive change over 18 months across multiple assessments in a cohort of participants ranging from cognitively normal (CN) controls through AD patients with moderate dementia. In addition, we evaluated the relative impact of defining low tau versus high tau subgroups using distribution-based and empirically derived cut points across different tau PET ROIs for predicting clinically meaningful longitudinal cognitive change on each assessment as defined by established thresholds for minimal clinically important differences (MCIDs).

## Methods

### Study design

Baseline and longitudinal data from an observational study evaluating longitudinal change in [^18^F]GTP1 tau PET in CN and AD participants (GN30009; NCT02640092) were analyzed. Baseline data from this study have been included elsewhere [3, 21–23].

### Participants

A total of 67 CN and AD participants between 50 and 85 years of age were enrolled from 11 research centers between December 2015 and November 2017. Data from participants with baseline neuroimaging and cognitive assessments and at least one post-baseline cognitive assessment were analyzed. Inclusion criteria for the CN group (*n* = 10) included no subjective or objective cognitive concerns, global Clinical Dementia Rating (CDR) [24] of 0, and Mini-Mental State Examination (MMSE) [25] of 28–30. In order to capture a broader range of tau PET imaging in the CN group, we included both Aβ PET-positive (by visual read [26]; *n* = 3) and Aβ PET-negative (*n* = 7) participants. In contrast, all AD participants had positive Aβ PET scans as well as brain MRIs without significant non-AD disease likely to contribute to cognitive impairment. Prodromal AD participants (*n* = 26) met NIA-AA criteria for mild cognitive impairment (MCI) [27] and had global CDRs of 0.5 and MMSEs of 24–30. Mild (*n* = 16) and moderate (*n* = 15) AD participants met NIA-AA criteria for probable AD dementia [28]. Mild AD participants had global CDRs of 0.5 or 1 and MMSEs of 22–30. Prodromal and mild AD were differentiated per investigators’ application of NIA-AA criteria for MCI versus dementia. Moderate AD participants had global CDRs of 0.5, 1, or 2 and MMSEs of 16–21.

This study was approved by each center’s Institutional Review Board and conducted in accordance with International Conference on Harmonization E6 Guidelines for Good Clinical Practice. All participants and/or their legally authorized representatives provided written informed consent.

### Neuroimaging

[^18^F]GTP1 preparation and PET were performed centrally (Invicro; New Haven, CT) as previously described [3]. Images were acquired with Siemens HR+ PET or Biograph 6 PET-CT cameras during a 30-min window 60 min post-injection after a mean (*SD*) bolus injection of 343 (31) MBq and reconstructed with an iterative reconstruction algorithm (OSEM 4 iterations, 16 subsets) and a post hoc 5-mm Gaussian filter. Individual PET frames were motion-corrected and average [^18^F]GTP1 images were created and co-registered to MRI, which was then spatially normalized to standard Montreal Neurological Institute space with SPM12 (www.fil.ion.ucl.ac.uk/spm/software/spm12). Normalization parameters were applied to the corresponding average [^18^F]GTP1 image. Composite ROIs included whole cortical gray matter (WCG), an AD-signature temporal ROI [20], and hierarchical in vivo Braak (I/II, III/IV, V/VI) [29, 30] that correspond to neuropathological NFT staging [31]. MRI tissue segmentation was performed to define the cortical gray matter, and the Hammers atlas [32] was used to define ROIs as previously described [3]. The temporal ROI included the hippocampus, amygdala, anterior medial temporal, anterior lateral temporal, parahippocampal and ambient gyri, middle and inferior temporal gyrus, and fusiform gyrus ROIs from the Hammers atlas. [^18^F]GTP1 standardized uptake value ratios (SUVRs) were calculated using bilateral inferior cerebellar gray as reference [3]. PET data were not partial volume corrected.

[^18^F]florbetapir was prepared at commercial facilities and Aβ PET was performed at individual sites per manufacturer instructions (Eli Lilly; Indianapolis, IN). Aβ PET SUVRs for a global cortical ROI were calculated using the whole cerebellum as reference.

MRI was performed at individual sites on 1.5T or 3T scanners. 3D sagittal T1-weighted MPRAGE sequences were collected for volumetric analyses and PET image processing as previously described [21]. Cortical volume was quantified using Freesurfer 6.0 (http://freesurfer.net) measurements of cortical segmentation for whole cortical gray matter and adjusted for intracranial volume.

### Neuropsychological testing

Cognitive assessments at baseline and 6-, 12-, and 18-month post-baseline visits included the MMSE, CDR, 13-item version of the Alzheimer’s Disease Assessment Scale-Cognitive Subscale (ADAS-Cog13) [33], and Repeatable Battery for the Assessment of Neuropsychological Status (RBANS) [34]. The MMSE and ADAS-Cog13 were analyzed using total scores, the CDR was analyzed using the Sum of Boxes (CDR-SB), and the RBANS was analyzed using Total Index scores.

### Statistical methods

Primary statistical analyses were performed with R (v.3.3.2) [35]. Baseline comparisons between diagnostic groups were conducted using one-way ANOVAs for continuous measures and chi-squared tests for categorical variables. Post hoc analyses were performed using Tukey’s test for continuous measures and Bonferroni correction for categorical variables to correct for multiple comparisons between diagnostic groups. Longitudinal cognitive changes were analyzed using estimated slopes derived from both simple linear and linear mixed effects models. For the simple linear model, a regression line was fit to each participant’s cognitive scores as a function of years post-baseline to obtain an annualized slope estimate. The linear mixed effects model estimated annualized change from baseline as a function of diagnostic cohort and time post-baseline, incorporating random effects for participants. Initially, univariate associations between baseline neuroimaging and cognitive change were assessed using Spearman correlations and annualized slopes derived from the simple linear models. These univariate analyses were then confirmed with separate multiple linear mixed effects regression models to assess associations between annualized rates of change on specific assessments and baseline [^18^F]GTP1 SUVR (using each composite [^18^F]GTP1 ROI) versus other relevant covariates: [^18^F]florbetapir SUVR, cortical volume, baseline cognitive performance, and age (all variables continuous).

**Fig. 1:**
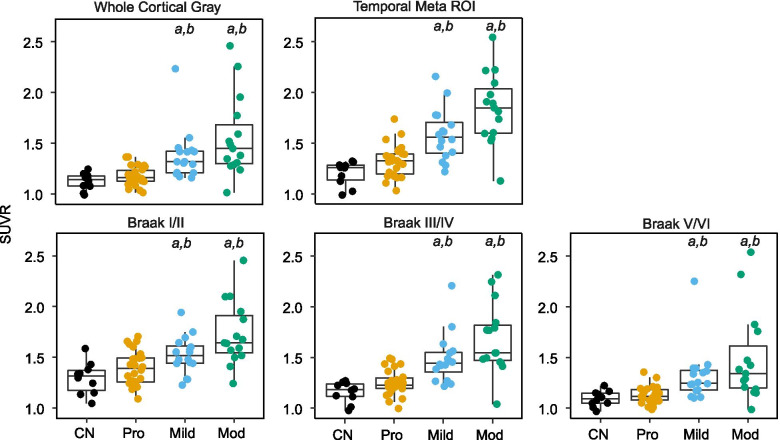
Baseline [^18^F]GTP1 standardized uptake value ratios (SUVRs) in the temporal, whole cortical gray, temporal, and Braak regions of interest (ROIs) in the cognitively normal (CN) and prodromal (Pro), mild (Mild), and moderate (Mod) AD groups. ^a^*p* < 0.05 vs. CN; ^b^*p* < 0.05 vs. Pro

Thresholds for elevated tau on [^18^F]GTP1 PET were determined via two methods. The first was a distribution-based approach focused on distinguishing CN and AD participants. Given the small size of the CN cohort (*n* = 10) and slightly skewed SUVR distribution (Fig. 1), the upper bound of SUVR range in the WCG (SUVR = 1.245) or temporal (SUVR = 1.325) ROIs in the CN group was used to dichotomize AD participants as T- versus T+. While these distribution-based cutoffs for [^18^F]GTP1 are based on limited data, the temporal ROI threshold is similar to those previously calculated from larger datasets for flortaucipir (1.36, *95% CI *1.34–1.40), MK-6240 (1.36, *95% CI* 1.16–1.49), and RO948 (1.34, *95% CI* 1.24–1.39) [36]. The second was an empirical approach that dichotomized AD participants into decliners versus non-decliners based on whether they experienced MCIDs on cognitive assessments during longitudinal follow-up. MCIDs are patient-centered thresholds that represent the smallest changes on outcome measures that are meaningful to patients at an individual level [37]. MCIDs for clinical decline have been identified as a 3 point decrease on the MMSE [38, 39], a 1 point increase on the CDR-SB [40], or a 3 point increase on the ADAS-Cog [41]. While an MCID has been proposed for the RBANS Total Index [42], it was not included in these analyses, as subsequent work has questioned its utility [43]. Receiver operating characteristic (ROC) analyses with Youden’s Index were used to determine [^18^F]GTP1 SUVR thresholds that optimally discriminated between participants whose decline did or did not meet MCID criteria on the MMSE, CDR-SB, or ADAS-Cog13. Logistic regression analyses were subsequently performed to calculate odds ratios (*OR*s) for each distribution-based or empiric SUVR cut point reflecting the increase in odds of cognitive decline meeting MCID thresholds for AD participants classified as T+ versus those classified as T-.

## Results

### Baseline participant characteristics

Baseline data for each diagnostic group are shown in Table 1. Diagnostic groups were similar in age, racial/ethnic background, and gender distribution (*p*s > 0.1). A significantly higher proportion of APOE ε4 carriers were seen in the moderate AD subgroup relative to the CN group (*p* = 0.029). Progressively lower MMSE and RBANS scores and higher CDR-SB and ADAS-Cog13 scores were seen with increasing disease severity (*p*s < 0.001). Lower proportions of AD participants had complete longitudinal cognitive data than the CN group, due to a combination of slightly greater attrition and, at later disease stages, incomplete ADAS-Cog13 and RBANS data due to the inability to complete more challenging subtests. Likewise, progressively higher [^18^F]GTP1 WCG SUVRs and lower cortical volumes were seen with increasing disease severity (*p*s < 0.001). Greater cortical Aβ PET SUVRs were seen in each AD subgroup relative to the CN group (*p*s < 0.05). Mean cortical Aβ PET SUVRs did not differ across AD subgroups (*p*s > 0.10).

**Table 1:** Baseline demographic, cognitive, and neuroimaging characteristics of study cohort

	CN (***n*** = 10)	Prodromal AD (***n*** = 26)	Mild AD (***n*** = 16)	Moderate AD (***n*** = 15)	***p***
Demographics					
Age	67.2 (6.2)	69.8 (6.9)	71.9 (4.9)	70.8 (7.0)	0.31
Sex (% male)	40%	46%	44%	53%	0.92
Race/ethnicity (% non-Hispanic white)	90%	96%	81%	87%	0.47
APOE status (% ε4+)	40%	78%	63%	93%^a^	0.023
MMSE					
Baseline	29.2 (0.8)	28.0 (1.5)	26.0 (2.9)^a,b^	16.9 (2.8)^a,b,c^	<0.001
% complete 18-month data	90%	85%	75%	73%	0.65
Average follow-up (days)	509.8 (122.9)	496.7 (128.9)	520.4 (82.0)	459.5 (141.9)	0.55
CDR-SB					
Baseline	0.0 (0.2)	1.6 (0.8)^a^	3.3 (1.8)^a,b^	6.4 (1.9)^a,b,c^	<0.001
% complete 18-month data	90%	85%	75%	73%	0.65
Average follow-up (days)	539.0 (124.3)	535.0 (127.2)	562.2 (76.6)	482.7 (181.4)	0.40
ADAS-Cog13					
Baseline	9.3 (5.0)	14.8 (5.5)	22.1 (6.8)^a,b^	40.3 (6.9)^a,b,c^	<0.001
% complete 18-month data	90%	85%	75%	67%	0.43
Average follow-up (days)	510.2 (123.1)	497.2 (129.2)	521.8 (83.4)	447.4 (142.3)	0.37
RBANS Total Index					
Baseline	93.0 (10.8)	85.1 (11.8)	73.2 (14.8)^a,b^	55.8 (9.6)^a,b,c^	<0.001
% complete 18-month data	90%	85%	44%^b^	27%^b^	<0.001
Average follow-up (days)	510.2 (123.1)	497.2 (129.2)	421.2 (165.7)	361.2 (166.4)^b^	0.031
Neuroimaging					
[^18^F]GTP1: WCG SUVR	1.12 (0.08)	1.18 (0.09)^a^	1.38 (0.26)^a,b^	1.55 (0.40)^a,b^	<0.001
[^18^F]florbetapir: cortical SUVR	1.18 (0.18)	1.37 (0.14)^a^	1.38 (0.12)^a^	1.41 (0.17)^a^	0.003
MRI: cortical volume (cm^3^)	429.7 (39.4)	444.5 (39.7)	407.8 (31.0)	372.4 (77.4)^a,b^	<0.001

Data are expressed as means (*SD*). *p*-values refer to overall one-way ANOVA or chi-square tests. ^a^*p* < 0.05 vs. CN; ^b^*p* < 0.05 vs. prodromal AD; ^c^*p* < 0.05 vs. mild AD. *Abbreviations*: *CN*, cognitively normal; *MMSE*, Mini-Mental State Examination; *CDR-SB*, Clinical Dementia Rating Sum of Boxes; *ADAS-Cog13*, 13-item version of the Alzheimer’s Disease Assessment Scale-Cognitive Subscale; *RBANS*, Repeatable Battery for the Assessment of Neuropsychological Status; *WCG*, whole cortical gray; *SUVR*, standardized uptake value ratio

Baseline [^18^F]GTP1 SUVRs in WCG, temporal, and hierarchical in vivo Braak ROIs progressively increased with disease severity (Fig. 1). Significant group effects were seen in all ROIs [WCG: *F*(3, 63) = 10.91, *p* < 0.001; temporal: *F*(3, 63) = 21.77, *p* < 0.001; Braak I/II: *F*(3, 63) = 11.21, *p* < 0.001; Braak III/IV: *F*(3, 63) = 17.14, *p* < 0.001; Braak V/VI: *F*(3, 63) = 7.96, *p* < 0.001]. Post hoc analyses (adjusted for multiple comparisons) across all ROIs indicated [^18^F]GTP1 SUVRs in the CN group did not significantly differ from the prodromal AD group (*p*s > 0.10), but were significantly lower than the mild and moderate AD groups (*p*s < 0.05). [^18^F]GTP1 SUVRs in prodromal AD were significantly lower than in moderate AD in all ROIs (*p*s < 0.05) and in mild AD in all ROIs (*p*s < 0.05) except Braak I/II (*p* = 0.12). Similar [^18^F]GTP1 SUVRs were seen between mild and moderate AD groups across all ROIs (*p*s > 0.05).

### Longitudinal change on cognitive indices

**Fig. 2:**
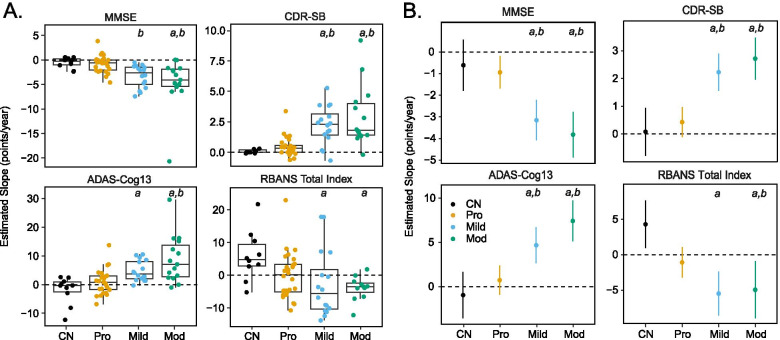
Annualized longitudinal cognitive change using estimated slopes derived from **A** simple linear and **B** linear mixed effect models on the Mini-Mental State Exam (MMSE), Clinical Dementia Rating Sum of Boxes (CDR-SB), 13-item version of the Alzheimer’s Disease Assessment Scale-Cognitive Subscale (ADAS-Cog13), and Repeatable Battery for the Assessment of Neuropsychological Status (RBANS) Total Index in the cognitively normal (CN) and prodromal (Pro), mild (Mild), and moderate (Mod) AD groups. Error bars indicate a 95% confidence interval. ^a^*p* < 0.05 vs. CN; ^b^*p* < 0.05 vs. Pro

Longitudinal change on the MMSE, CDR-SB, ADAS-Cog13, and RBANS was determined via estimated slopes derived from simple linear modeling (Fig. 2A) and confirmed via linear mixed effects modeling (Fig. 2B). One-sample *t*-tests performed on estimated slopes derived from simple linear modeling indicated that MMSE, CDR-SB, and ADAS-Cog13 scores remained stable in the CN group (*p*s > 0.05). However, on the RBANS, the CN group improved across assessments (*p* = 0.035), primarily between baseline and 6-month timepoints, likely due to practice effects [44, 45], and remained stable thereafter (Supplemental Fig. 1). The prodromal AD group declined on the CDR-SB (*p* = 0.010) and MMSE (*p* = 0.039), but not on the ADAS-Cog13 or RBANS (*p* > 0.05). The mild AD group declined on the MMSE, CDR-SB, and ADAS-Cog13 (*p*s < 0.001) but not the RBANS (*p* > 0.05). The moderate AD group declined on all indices (*p*s < 0.006). One-way ANOVAs indicated significant group effects on the MMSE [*F*(3,63)=7.74, *p* < 0.001], CDR-SB [*F*(3,63) = 13.07, *p* < 0.001], ADAS-Cog13 [*F*(3,63) = 10.65, *p* < 0.001], and RBANS [*F*(3,59) = 3.51, *p* = 0.021]. Post hoc analyses indicated that more rapid declines occurred in the mild AD group relative to the CN (CDR-SB, ADAS-Cog13, RBANS) and prodromal AD (MMSE, CDR-SB) groups (*p*s < 0.05). Likewise, the moderate AD group exhibited steeper declines than the CN (MMSE, CDR-SB, ADAS-Cog13, RBANS) and prodromal AD (MMSE, CDR-SB, ADAS-Cog13) groups (*p*s < 0.05).

Linear mixed effects modeling (Fig. 2B) revealed significant effects of assessment visit [MMSE: *F*(1,175) = 74.57, *p* < 0.001; CDR-SB: *F*(1,175) = 58.99, *p* < 0.001; ADAS-Cog13: *F*(1,174) = 31.81, *p* < 0.001; RBANS: *F*(1,152) = 5.74, *p* = 0.018], group [MMSE: *F*(3,63) = 98.26, *p* < 0.001; CDR-SB: *F*(3,63) = 52.28, *p* < 0.001; ADAS-Cog13: *F*(3,63) = 63.90, *p* < 0.001; RBANS: *F*(3,60) = 31.73, *p* < 0.001], and group x visit interaction [MMSE: *F*(3,175) = 10.24, *p* < 0.001; CDR-SB: *F*(3,175) = 13.12, *p* < 0.001; ADAS-Cog13: *F*(3,174) = 11.04, *p* < 0.001; RBANS: *F*(3,152) = 6.91, *p* < 0.001]. Post hoc analyses (adjusted for multiple comparisons) revealed more rapid declines in the mild AD group relative to the CN (MMSE, CDR-SB, ADAS-Cog13, RBANS) and prodromal AD (MMSE, CDR-SB, ADAS-Cog13) groups (*p*s < 0.05). Likewise, the moderate AD group exhibited steeper declines than the CN (MMSE, CDR-SB, ADAS-Cog13, RBANS) and prodromal AD (MMSE, CDR-SB, ADAS-Cog13) groups (*p*s < 0.05). CN and prodromal AD participants were more likely to have complete longitudinal cognitive data than mild or moderate AD participants (Table 1), due to increased dropout and/or inability to complete one or more ADAS-Cog13 or RBANS subtest in the latter two groups over the course of study due to disease progression.

### Continuous associations between baseline [^18^F]GTP1 SUVR and longitudinal change on cognitive indices

**Fig. 3:**
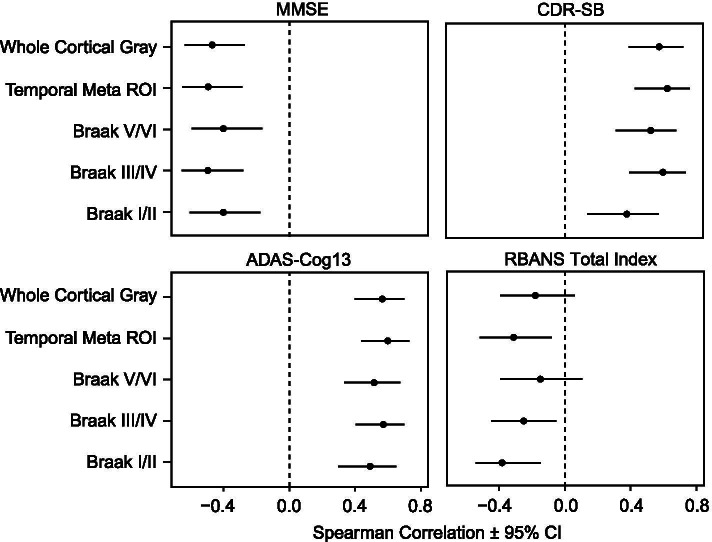
Forest plots illustrating Spearman correlations of baseline [^18^F]GTP1 standardized uptake value ratios across different regions of interest versus annualized change scores calculated via estimated slopes on the Mini-Mental State Exam (MMSE), Clinical Dementia Rating Sum of Boxes (CDR-SB), 13-item version of the Alzheimer’s Disease Assessment Scale-Cognitive Subscale (ADAS-Cog13), and Repeatable Battery for the Assessment of Neuropsychological Status (RBANS) Total Index

Univariate associations between baseline [^18^F]GTP1 SUVR across different ROIs versus annualized cognitive change scores estimated via simple linear models are illustrated in Fig. 3 and Supplemental Fig. 2. Spearman correlations indicated that a greater cognitive decline correlated with higher baseline [^18^F]GTP1 SUVR values in the WCG, temporal, Braak I/II, Braak III/IV, and Braak V/VI ROIs (*p*s < 0.05), with the exception of the RBANS in WCG (*r*_*s*_=−0.18, *p* = 0.153) and Braak V/VI (*r*_*s*_ = −0.15, *p* = 0.238) ROIs. Across these ROIs, correlations between baseline [^18^F]GTP1 SUVRs and longitudinal change on the MMSE, CDR-SB, and ADAS-Cog13 remained largely consistent. Correlational analyses of baseline [^18^F]GTP1 SUVR versus cognitive change within diagnostic subgroups were limited by small sample sizes and failed to reveal consistent patterns (Supplemental Table 1).

Multivariate regression analyses that included all participants (Table 2) were performed to investigate associations between baseline [^18^F]GTP1 SUVR and subsequent cognitive change relative to other potential prognostic factors (baseline cognitive performance on each measure, participant age, [^18^F]florbetapir SUVR, and cortical volume as measured by MRI). WCG [^18^F]GTP1 SUVR was independently associated with annualized cognitive change across all four measures. Neither cortical volume nor [^18^F]florbetapir SUVR were significantly associated with declines on any measure. Similar results emerged with analyses limited to data from pooled AD participants (prodromal, mild, and moderate; Supplemental Table 2), suggesting that our overall findings were not primarily driven by inclusion of the CN group in the analyses. In analogous multivariate linear regression analyses performed using other ROIs, particularly the temporal and Braak I/II ROIs, less robust associations were seen with baseline [^18^F]GTP1 SUVRs and subsequent cognitive performance (Supplemental Tables 2 and 3). The size of the study cohort was not large enough to perform reliable mediation analyses of the relationship between baseline Aβ and tau PET on the subsequent longitudinal decline.

**Table 2:** Linear mixed effects models assessing change on cognitive indices from baseline incorporating demographic, baseline cognitive, and imaging variables (using the WCG ROI and all participants). Partial regression coefficients (*B*), standard errors (*SE*), and *t* statistics are reported for each predictor; *r*^2^ values are reported for each linear mixed effect model

WCG ROI	MMSE ***r***^**2**^ = 0.38		CDR-SB ***r***^**2**^ = 0.37		ADAS-Cog13 ***r***^**2**^ = 0.55		RBANS ***r***^**2**^ = 0.22	
Covariate (units)	***B*** (***SE***)	***p***	***B*** (***SE***)	***p***	***B*** (***SE***)	***p***	***B*** (***SE***)	***p***
Baseline score (1 point)	0.07 (0.05)	0.217	0.20 (0.07)	***0.008***	0.07 (0.04)	0.157	−0.01 (0.06)	0.895
Age (10 years)	−0.68 (0.35)	0.056	0.76 (0.23)	***0.001***	1.35 (0.68)	***0.049***	−2.15 (1.17)	0.069
[^18^F]florbetapir (cortex; 0.1 SUVR)	−0.05 (0.15)	0.72	-0.09 (0.10)	0.380	0.03 (0.29)	0.926	0.11 (0.52)	0.846
MRI (cortical volume; cm^3^)	0.00 (0.01)	0.961	0.00 (0.00)	0.361	−0.02 (0.01)	0.107	−0.04 (0.02)	0.077
[^18^F]GTP1 (WCG; 0.1 SUVR)	−0.28 (0.12)	***0.015***	0.16 (0.08)	***0.043***	0.65 (0.23)	***0.006***	−1.62 (0.64)	***0.013***

*Abbreviations*: *MMSE*, Mini-Mental State Examination; *CDR-SB*, Clinical Dementia Rating Sum of Boxes; *ADAS-Cog13*, 13-item version of the Alzheimer’s Disease Assessment Scale-Cognitive Subscale; *RBANS*, Repeatable Battery for the Assessment of Neuropsychological Status Total Index; *WCG*, whole cortical gray; *SUVR*, standardized uptake value ratio

### Distribution-based [^18^F]GTP1 SUVR cut points and longitudinal change on cognitive indices

**Fig. 4:**
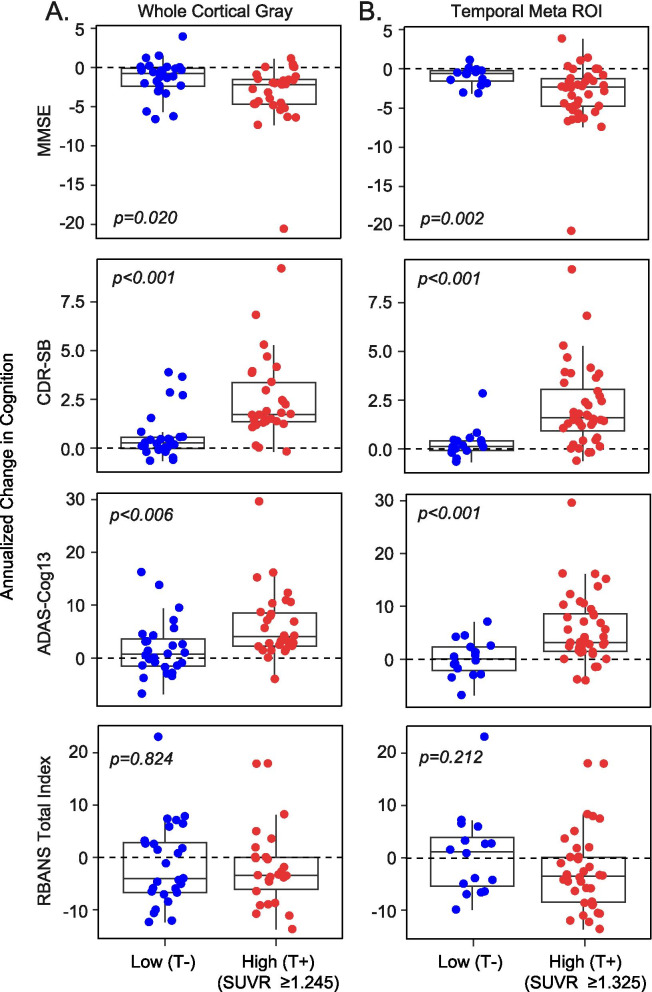
Boxplots of annualized rates of change on the Mini-Mental State Exam (MMSE), Clinical Dementia Rating Sum of Boxes (CDR-SB), 13-item version of the Alzheimer’s Disease Assessment Scale-Cognitive Subscale (ADAS-Cog13), and Repeatable Battery for the assessment of Neuropsychological Status (RBANS) Total Index for study participants dichotomized by distribution-based [^18^F]GTP1 SUVR cutoffs of **A** 1.245 in the whole cortical gray ROI or **B** 1.325 in the temporal ROI

When distribution-based SUVR thresholds were used to subgroup AD participants as T- versus T+, a greater proportion were classified as T+ with the temporal ROI cut point (SUVR ≥ 1.325; prodromal 56%, mild 84%, moderate 88%) than with the WCG ROI cut point (SUVR = 1.245; prodromal 26%, mild 74%, moderate 81%). With the WCG ROI cutoff (Fig. 4A), T+ participants had a more rapid decline than T- participants on the MMSE, CDR-SB, and ADAS-Cog13 (*p*s < 0.05), but not on the RBANS (*p* = 0.824). Likewise, with the temporal ROI cut point (Fig. 4B), T+ participants had more rapid decline than T- participants on the MMSE, CDR-SB, and ADAS-Cog13 (*p*s ≤ 0.005), but not on the RBANS (*p* = 0.212). Similar results were seen when distribution-based SUVR cut points were used for the Braak I/II, III/IV, and V/VI ROIs (Supplemental Fig. 3).

### Empirical [^18^F]GTP1 SUVR cut points derived from MCIDs

AD participants were dichotomized as decliners versus non-decliners on each assessment based on whether they experienced declines meeting MCID thresholds during longitudinal follow-up. Across assessments (Supplemental Table 4), the temporal ROI distribution-based cut point yielded good sensitivity (0.89–0.91) but poor specificity (0.43–0.59) for predicting meaningful decline, whereas the WCG ROI distribution-based cut point resulted in a greater balance between sensitivity (0.67–0.74) and specificity (0.60–0.82).

**Fig. 5:**
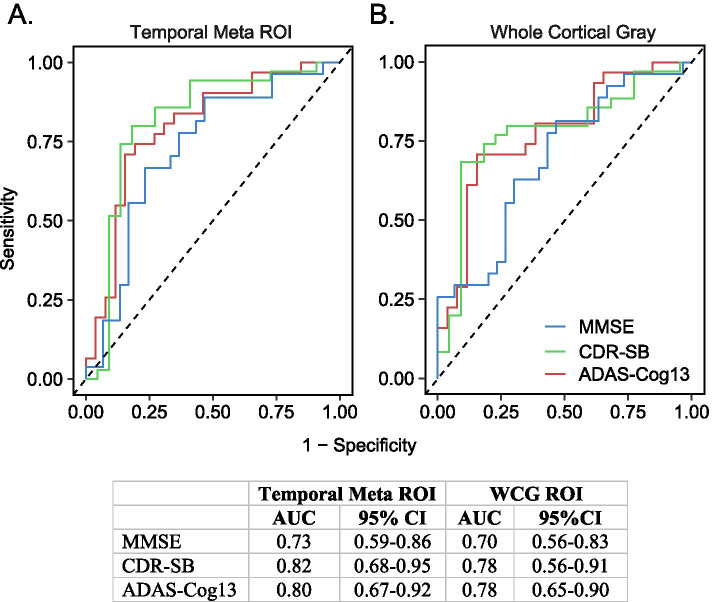
Receiver operating characteristic curves for **A** temporal meta ROI and **B** whole cortical gray (WCG) ROI [^18^F]GTP1 SUVR for distinguishing between progressors and non-progressors on the Mini-Mental State Exam (MMSE), Clinical Dementia Rating Sum of Boxes (CDR-SB), and 13-item version of the Alzheimer’s Disease Assessment Scale-Cognitive Subscale (ADAS-Cog13)

ROC analyses were used to optimize empirically determined tau PET cut points for distinguishing between decliners versus non-decliners for each cognitive assessment (Fig. 5). Area under the curve (AUC) values were similar for mean [^18^F]GTP1 SUVR values in the temporal (0.73–0.82) and WCG (0.70–0.78) ROIs. Similar AUC values were obtained from ROC analyses with mean [^18^F]GTP1 SUVR values in Braak III/IV ROI, but lower AUC values were seen in the Braak I/II and V/VI ROIs (Supplemental Fig. 4). Optimized empirical [^18^F]GTP1 SUVR cut points ranged from 1.406 to 1.533 (sensitivity 0.67–0.80; specificity 0.77–0.85) in the temporal ROI and 1.194–1.286 (sensitivity 0.69–0.81; specificity 0.53–0.91) in the WCG ROI (Supplemental Table 4). For the Braak ROIs, the optimal cut points were highest in Braak I/II (1.497–1.562) and progressively lower in the Braak III/IV (1.269–1.412) and Braak V/VI (1.184–1.248) ROIs (Supplemental Table 4).

### Odds ratios for clinical decline exceeding MCID thresholds with distribution-based and empirical [^18^F]GTP1 SUVR cut points

Participants with baseline SUVRs exceeding distribution-based cutoffs in either the WCG or temporal ROIs were significantly more likely to experience subsequent clinical decline that exceeded MCID-defined thresholds on the MMSE (WCG: *OR* = 3.00; *p* = 0.047, temporal: *OR* = 6.12, *p* = 0.011), CDR-SB (WCG: *OR* = 13.00, *p* < 0.001; temporal: *OR* = 15.41, *p* < 0.001), and ADAS-Cog13 (WCG: *OR* = 5.50, *p* = 0.003; temporal: *OR* = 9.33, *p* = 0.002). Similar results were seen when distribution-based SUVR cut points were used for the Braak I/II, III/IV, and V/VI ROIs (Supplemental Table 5). As expected, empirical SUVR cut points derived from MCID-defined thresholds yielded higher *OR*s on the MMSE (WCG: *OR* = 5.03, *p* = 0.009; temporal: *OR* = 6.57, *p* = 0.002), CDR-SB (WCG: *OR* = 21.82, *p* < 0.001; temporal: *OR* = 18.00, *p* < 0.001), and ADAS-Cog13 (WCG: *OR* = 13.44, *p* < 0.001; temporal: *OR* = 13.44, *p* < 0.001). Similar results were seen when empirical cut points were used for the Braak I/II, III/IV, and V/VI ROIs (Supplemental Table 5).

## Discussion

Our data indicate that higher baseline [^18^F]GTP1 SUVRs are independently associated with steeper cognitive declines across a spectrum of AD severity and across a range of different cognitive measures. These results establish [^18^F]GTP1 tau PET as a potential prognostic imaging biomarker in AD and complement prior work from our group demonstrating that [^18^F]GTP1 tau PET imaging differentiates between AD cohorts of different severity [3] and exhibits significant cross-sectional correlations with cognitive performance [21]. These results are consistent with previous studies supporting the prognostic potential of other tau PET tracers, including [^18^F]flortaucipir [10–13, 17], [^18^F]MK-6240 [15], and [^18^F]RO948 [17]. Together, these findings reaffirm the proposed temporal relationship between tau pathology and cognitive decline [46] and establish the potential of tau PET for identifying AD patients in clinical practice who are at risk for more rapid cognitive decline and for stratification and/or enrichment of participant selection in therapeutic clinical trials in AD. The commonality of results across different tau PET tracers suggests similar labeling of tau NFTs [4] and similar prognostic value.

Prior studies examining cross-sectional tau PET and cognitive decline have focused on specific ROIs, including inferior temporal cortex [12, 13], hippocampus [12], and entorhinal cortex [12, 15] in preclinical AD cohorts, or composite ROIs weighted towards occipital, parietal, and lateral and posterior temporal cortices [11] or temporal and parietal cortices [16] in broader AD cohorts. In our sample, which falls into the latter category, similar correlations between baseline [^18^F]GTP1 PET and subsequent decline were seen irrespective of the ROIs examined (Fig. 3), arguing against the overall superiority of any specific composite ROI. These results likely reflect that much of the [^18^F]GTP1 signal in the pre-specified ROIs (except Braak V/VI) is driven by temporal lobe tau [3] given the hierarchical pattern of tau pathology in AD (i.e., significant temporal NFT deposition prior to widespread extra-temporal NFT accumulation [31]). Therefore, while specific temporal ROIs may be critical for predicting cognitive trajectories in preclinical AD, when extra-temporal NFTs are relatively scarce [12–15], more global ROIs may be sufficient at later disease stages.

Correlations between baseline [^18^F]GTP1 SUVRs and cognitive decline were largely consistent across assessments, though less robust correlations were seen with the RBANS. This result may be attributable to greater heterogeneity in longitudinal RBANS data relative to other assessments, floor effects in moderate AD, and/or test-retest effects masking cognitive decline in CN or prodromal AD [44, 45]. Our results are concordant with prior reports of correlations between baseline tau PET and cognitive decline across a variety of composite measures [11, 13, 15–17]. Studies in participants with normal cognition or MCI have primarily shown correlations between tau PET and declines in episodic memory [10, 12], which may be indicative of the greater sensitivity of memory tests for cognitive change in preclinical and prodromal AD. This overall pattern of results suggests that tau PET is broadly prognostic for subsequent decline across a spectrum of longitudinal assessments in AD.

Another approach that may be more applicable to patient care and clinical trials in AD is determining whether threshold-based classification of individuals as having low versus high tau burden [20] has utility for predicting rates of future cognitive decline. When using thresholds optimized for AD diagnosis, high tau PET participants demonstrate steeper rates of cognitive decline, both in the current study and prior reports [15]. However, when such thresholds were used to predict clinically meaningful cognitive decline, they exhibited excellent sensitivity but mediocre specificity, particularly with the temporal ROI. Empirically defined thresholds for optimizing sensitivity and specificity for clinically meaningful decline yielded higher SUVR cutoffs, though increased specificity was offset by reduced sensitivity. Both distribution-based and empirically defined cut points for low versus high tau burden identified participants at higher risk of clinical progression that exceeded MCIDs, supporting the potential utility of these approaches. Given the continuous distribution of [^18^F]GTP1 SUVR with increasing disease severity [3, 21], these data suggest that thresholds for distinguishing high versus low tau PET are likely to depend on the specific questions of interest. In contrast, for Aβ PET, which has a more bimodal distribution between AD patients and controls [47], the same threshold for high versus low Αβ pathology may be useful for both diagnosis and prognosis [48].

### Limitations

While our results confirm and extend prior work supporting tau PET as a prognostic biomarker in AD [10–12, 15–17], a number of factors may limit their interpretation. The size of the study cohort was relatively small, and there were likely too few participants within each AD subgroup to detect specific prognostic relationships between tau PET and cognitive decline at different disease stages. Nevertheless, the prognostic association seen between [^18^F]GTP1 SUVR and cognitive decline is concordant with a larger study with [^18^F]flortaucipir [11]. Likewise, the small size of our CN cohort may have limited our ability to demonstrate statistically significant differences in [^18^F]GTP1 SUVR relative to the prodromal AD cohort. Furthermore, the slightly skewed distribution of baseline [^18^F]GTP1 SUVRs in the CN group precluded more direct comparisons of distribution-derived tau PET positivity thresholds to other tracers [15, 20]. Higher rates of incomplete longitudinal neuropsychological data collection were seen amongst participants with more advanced AD, particularly for the RBANS. As such, our results may represent an underestimate of the correlation between baseline [^18^F]GTP1 SUVRs and subsequent cognitive decline. Finally, as previously noted [21], we did not collect participants’ level of education so we were unable to include it in our analyses. However, in prior multivariate analyses that have included education, the association between tau PET and cognitive decline remains robust [10, 16].

## Conclusions

Compelling observational data from this study and others [10–12, 15–17] support a role for tau PET in patient selection/stratification in therapeutic clinical trials in AD. [^18^F]GTP1 tau PET is included in trials with gantenerumab (anti-Aβ antibody; NCT03443973, NCT03444870), semorinemab (anti-tau antibody; NCT03289143, NCT03828747), and bepranemab (anti-tau antibody; NCT04867616). Forthcoming data from these larger studies may confirm the utility of [^18^F]GTP1 PET for predicting cognitive decline and/or response to anti-Aβ and/or anti-tau interventions.

## Supplementary Information

**Additional file 1: Supplemental Table 1.** Spearman correlations between baseline [^18^F]GTP1 standardized uptake value ratios and annualized slopes of longitudinal change on cognitive assessments across diagnostic groups and regions of interest. Abbreviations: ROIs, regions of interest; MMSE, Mini-Mental State Examination; CDR-SB, Clinical Dementia Rating Sum of Boxes; ADAS-Cog13, 13-item version of the Alzheimer’s Disease Assessment Scale-Cognitive Subscale; RBANS, Repeatable Battery for the Assessment of Neuropsychological Status; CN, cognitively normal; WCG, whole cortical gray. **Supplemental Table 2.** Linear mixed effects models assessing change on cognitive indices from baseline incorporating demographic, baseline cognitive, and imaging variables (using only AD participants). Partial regression coefficients (B), standard errors (SE), and *p* values are reported for each predictor; *r*^2^ values are reported for each linear mixed effect model. Abbreviations: MMSE, Mini-Mental State Examination; CDR-SB, Clinical Dementia Rating Sum of Boxes; ADAS-Cog13, 13-item version of the Alzheimer’s Disease Assessment Scale-Cognitive Subscale; RBANS, Repeatable Battery for the Assessment of Neuropsychological Status; WCG, whole cortical gray. **Supplemental Table 3.** Linear mixed effects models assessing change on cognitive indices from baseline incorporating demographic, baseline cognitive, and imaging variables (using temporal, Braak I/II, Braak III/IV, and Braak V/VI ROIs and all participants). Partial regression coefficients (B), standard errors (SE), and *p* values are reported for each predictor; *r*^2^ values are reported for each linear mixed effect model. Abbreviations: MMSE, Mini-Mental State Examination; CDR-SB, Clinical Dementia Rating Sum of Boxes; ADAS-Cog13, 13-item version of the Alzheimer’s Disease Assessment Scale-Cognitive Subscale; RBANS, Repeatable Battery for the Assessment of Neuropsychological Status; WCG, whole cortical gray. **Supplemental Table 4.** Sensitivity and specificity of [^18^F]GTP1 standardized uptake value ratio (SUVR) cut points for distinguishing between decliners and non-decliners. Abbreviations: MMSE, Mini-Mental State Examination; CDR-SB, Clinical Dementia Rating Sum of Boxes; ADAS-Cog13, 13-item version of the Alzheimer’s Disease Assessment Scale-Cognitive Subscale; ROI, region of interest; WCG, whole cortical gray. **Supplementary Table 5.** Odds ratios (ORs) for participants with prodromal, mild, or moderate AD experiencing cognitive decline that meets Minimal Clinically Important Differences (MCIDs) for participants with [^18^F]GTP1 SUVRs above specified cut points in specified ROIs. Abbreviations: MMSE, Mini-Mental State Examination; CDR-SB, Clinical Dementia Rating Sum of Boxes; ADAS-Cog13, 13-item version of the Alzheimer’s Disease Assessment Scale-Cognitive Subscale; ROI, region of interest; WCG, whole cortical gray.

**Additional file 2: Supplemental Figure 1.** Longitudinal change from baseline at each assessment timepoint using a mixed model repeated measure analysis on the Mini-Mental State Exam (MMSE), Clinical Dementia Rating Sum of Boxes (CDR-SB), 13-item version of the Alzheimer’s Disease Assessment Scale-Cognitive Subscale (ADAS-Cog13), and Repeatable Battery for the Assessment of Neuropsychological Status (RBANS) Total Index. SE: standard error; BL: baseline; W26: Week 26 visit; W52: Week 52 visit; W78: Week 78 visit.

**Additional file 3: Supplemental Figure 2.** Scatterplots of baseline [^18^F]GTP1 SUVR versus annualized change scores calculated via estimated slopes on the Mini-Mental State Exam (MMSE), Clinical Dementia Rating Sum of Boxes (CDR-SB), 13-item version of the Alzheimer’s Disease Assessment Scale-Cognitive Subscale (ADAS-Cog13), and Repeatable Battery for the Assessment of Neuropsychological Status (RBANS) Total Index in the cognitively normal (CN) and prodromal (Pro), mild (Mild), and moderate (Mod) AD groups in the (A) whole cortical gray, (B) temporal, (C) Braak I/II, (D) Braak III/IV, and (E) Braak V/VI ROIs.

**Additional file 4: Supplemental Figure 3.** Boxplots of annualized rates of change on the Mini-Mental State Exam (MMSE), Clinical Dementia Rating Sum of Boxes (CDR-SB), 13-item version of the Alzheimer’s Disease Assessment Scale-Cognitive Subscale (ADAS-Cog13), and Repeatable Battery for the Assessment of Neuropsychological Status (RBANS) Total Index for study participants dichotomized by distribution-based [^18^F]GTP1 SUVR cutoffs of (A) 1.586 in the Braak I/II ROI, (B) 1.268 in the Braak III/IV ROI, or (C) 1.232 in the Braak V/VI ROI.

**Additional file 5: Supplemental Figure 4.** Receiver Operating Characteristic curves for (A) Braak I/II, (B) Braak III/IV, and (C) Braak V/VI ROI [^18^F]GTP1 SUVRs for distinguishing between progressors and non-progressors on the Mini-Mental State Exam (MMSE), Clinical Dementia Rating Sum of Boxes (CDR-SB), and 13-item version of the Alzheimer’s Disease Assessment Scale-Cognitive Subscale (ADAS-Cog13).

## Data Availability

Qualified researchers may request access to individual patient-level data through the clinical study data request platform (https://vivli.org/). Further details on Roche’s criteria for eligible studies are available here: https://vivli.org/members/ourmembers/. For further details on Roche’s Global Policy on the Sharing of Clinical Information and how to request access to related clinical study documents, see https://www.roche.com/research_and_development/who_we_are_how_we_work/clinical_trials/ our_commitment_to_data_sharing.htm

## Abbreviations

AD: Alzheimer’s disease
ADAS-Cog13: Alzheimer’s Disease Assessment Scale-Cognitive Subscale
Aβ: β-amyloid
CDR: Clinical Dementia Rating
CN: Cognitively normal
MCI: Mild cognitive impairment
MCID: Minimal clinical important decline
MMSE: Mini-Mental State Exam
MRI: Magnetic resonance imaging
NIA-AA: National Institute on Aging-Alzheimer’s Association
NFTs: Neurofibrillary tangles
PET: Positron emission tomography
RBANS: Repeatable Battery for the Assessment of Neuropsychological Status
ROC: Receiver operating characteristic
ROI: Region of interest
*SD*: Standard deviation
SUVR: Standardized uptake value ratio
WCG: Whole cortical gray
