# Supplementary material for: Baseline [18F]GTP1 tau PET imaging is associated with subsequent cognitive decline in Alzheimer’s disease
Source: Alzheimers Res Ther. 2021 Dec 1;13:196. doi: 10.1186/s13195-021-00937-x (PMC8638526; doi:10.1186/s13195-021-00937-x)
Supplement: Supplementary file 1 — Additional file 1: Supplemental Table 1. Spearman correlations between baseline [18F]GTP1 standardized uptake value ratios and annualized slopes of longitudinal change on cognitive assessments across diagnostic groups and regions of interest. Abbreviations: ROIs, regions of interest; MMSE, Mini-Mental State Examination; CDR-SB, Clinical Dementia Rating Sum of Boxes; ADAS-Cog13, 13-item version of the Alzheimer’s Disease Assessment Scale-Cognitive Subscale; RBANS, Repeatable Battery for the Assessment of Neuropsychological Status; CN, cognitively normal; WCG, whole cortical gray. Supplemental Table 2. Linear mixed effects models assessing change on cognitive indices from baseline incorporating demographic, baseline cognitive, and imaging variables (using only AD participants). Partial regression coefficients (B), standard errors (SE), and p values are reported for each predictor; r2 values are reported for each linear mixed effect model. Abbreviations: MMSE, Mini-Mental State Examination; CDR-SB, Clinical Dementia Rating Sum of Boxes; ADAS-Cog13, 13-item version of the Alzheimer’s Disease Assessment Scale-Cognitive Subscale; RBANS, Repeatable Battery for the Assessment of Neuropsychological Status; WCG, whole cortical gray. Supplemental Table 3. Linear mixed effects models assessing change on cognitive indices from baseline incorporating demographic, baseline cognitive, and imaging variables (using temporal, Braak I/II, Braak III/IV, and Braak V/VI ROIs and all participants). Partial regression coefficients (B), standard errors (SE), and p values are reported for each predictor; r2 values are reported for each linear mixed effect model. Abbreviations: MMSE, Mini-Mental State Examination; CDR-SB, Clinical Dementia Rating Sum of Boxes; ADAS-Cog13, 13-item version of the Alzheimer’s Disease Assessment Scale-Cognitive Subscale; RBANS, Repeatable Battery for the Assessment of Neuropsychological Status; WCG, whole cortical gray. Supplemental Table 4. Sensitivity and specif [file 13195_2021_937_MOESM1_ESM.docx]

**Supplemental Table 1.** Spearman correlations between baseline [^18^F]GTP1 standardized uptake value ratios and annualized slopes of longitudinal change on cognitive assessments across diagnostic groups and regions of interest.

| **ROIs** | **MMSE** | | **CDR-SB** | | **ADAS-Cog13** | | **RBANS** | |
| --- | --- | --- | --- | --- | --- | --- | --- | --- |
|  | ***r_s_*** | ***p*** | ***r_s_*** | ***p*** | ***r_s_*** | ***p*** | ***r_s_*** | ***p*** |
| CN (n=10) |  |  |  |  |  |  |  |  |
| WCG | 0.34 | 0.34 | 0.03 | 0.93 | 0.12 | 0.76 | 0.27 | 0.45 |
| Temporal | 0.38 | 0.28 | 0.28 | 0.44 | 0.31 | 0.39 | 0.32 | 0.37 |
| Braak I/II | 0.16 | 0.66 | 0.24 | 0.51 | 0.64 | 0.06 | 0.02 | 0.97 |
| Braak III/IV | 0.24 | 0.50 | -0.09 | 0.80 | -0.04 | 0.92 | 0.43 | 0.22 |
| Braak V/VI | 0.35 | 0.33 | 0.15 | 0.67 | 0.14 | 0.71 | 0.16 | 0.66 |
| Prodromal AD (n=26) |  |  |  |  |  |  |  |  |
| WCG | -0.33 | 0.10 | 0.43 | 0.03 | 0.24 | 0.23 | -0.01 | 0.95 |
| Temporal | -0.27 | 0.17 | 0.36 | 0.07 | 0.19 | 0.36 | -0.34 | 0.09 |
| Braak I/II | -0.31 | 0.12 | 0.14 | 0.50 | 0.17 | 0.41 | -0.49 | 0.01 |
| Braak III/IV | -0.31 | 0.12 | 0.45 | 0.02 | 0.24 | 0.24 | -0.09 | 0.65 |
| Braak V/VI | -0.26 | 0.20 | 0.33 | 0.10 | 0.17 | 0.39 | 0.01 | 0.96 |
| Mild AD (n=16) |  |  |  |  |  |  |  |  |
| WCG | -0.27 | 0.32 | 0.32 | 0.23 | 0.29 | 0.27 | -0.19 | 0.49 |
| Temporal | -0.47 | 0.07 | 0.56 | 0.03 | 0.49 | 0.06 | -0.11 | 0.68 |
| Braak I/II | -0.38 | 0.14 | 0.19 | 0.49 | 0.31 | 0.25 | -0.40 | 0.12 |
| Braak III/IV | -0.31 | 0.25 | 0.36 | 0.17 | 0.34 | 0.20 | -0.22 | 0.40 |
| Braak V/VI | -0.14 | 0.60 | 0.27 | 0.32 | 0.26 | 0.34 | -0.25 | 0.35 |
| Moderate AD (n=15) |  |  |  |  |  |  |  |  |
| WCG | -0.07 | 0.80 | -0.25 | 0.39 | 0.28 | 0.31 | 0.74 | 0.01 |
| Temporal | 0.19 | 0.51 | -0.29 | 0.31 | 0.14 | 0.62 | 0.28 | 0.40 |
| Braak I/II | 0.26 | 0.34 | -0.47 | 0.09 | -0.06 | 0.82 | 0.23 | 0.50 |
| Braak III/IV | -0.01 | 0.96 | -0.26 | 0.37 | 0.18 | 0.53 | 0.53 | 0.10 |
| Braak V/VI | -0.05 | 0.86 | -0.20 | 0.50 | 0.32 | 0.25 | 0.78 | 0.01 |

Abbreviations: ROIs, regions of interest; MMSE, Mini-Mental State Examination; CDR-SB, Clinical Dementia Rating Sum of Boxes; ADAS-Cog13, 13-item version of the Alzheimer’s Disease Assessment Scale-Cognitive Subscale; RBANS, Repeatable Battery for the Assessment of Neuropsychological Status; CN, cognitively normal; WCG, whole cortical gray.**Supplemental Table 2.** Linear mixed effects models assessing change on cognitive indices from baseline incorporating demographic, baseline cognitive, and imaging variables (using only AD participants). Partial regression coefficients (B), standard errors (SE), and *p* values are reported for each predictor; *r*^2^ values are reported for each linear mixed effect model.

| **WCG ROI** | **MMSE**  ***r*^2^ = 0.37** | | **CDR-SB**  ***r*^2^ = 0.34** | | **ADAS-Cog13**  ***r*^2^ = 0.52** | | **RBANS**  ***r*^2^ = 0.23** | |
| --- | --- | --- | --- | --- | --- | --- | --- | --- |
| **Covariate (units)** | **B (SE)** | ***p*** | **B (SE)** | ***p*** | **B (SE)** | ***p*** | **B (SE)** | ***p*** |
| Baseline score  (1 point) | 0.05 (0.06) | 0.375 | 0.15 (0.09) | 0.094 | 0.07 (0.05) | 0.148 | -0.08 (0.06) | 0.193 |
| Age (10 years) | -0.85 (0.41) | ***0.042*** | 0.84 (0.27) | ***0.003*** | 1.08 (0.76) | 0.160 | -2.88 (1.20) | ***0.019*** |
| [^18^F]florbetapir  (cortex; 0.1 SUVR) | -0.03 (0.19) | 0.889 | -0.13 (0.12) | 0.300 | -0.08 (0.34) | 0.821 | 0.77 (0.56) | 0.173 |
| MRI cortical volume; (cm^3^) | 0.00 (0.01) | 0.924 | -0.01 (0.01) | 0.298 | -0.02 (0.01) | 0.264 | -0.03 (0.03) | 0.227 |
| [^18^F]GTP1  (WCG; 0.1 SUVR) | -0.30 (0.13) | ***0.021*** | 0.17 (0.09) | 0.052 | 0.65 (0.25) | ***0.012*** | -2.07 (0.67) | ***0.003*** |

| **Temporal ROI** | **MMSE**  ***r*^2^ = 0.36** | | **CDR-SB**  ***r*^2^ = 0.33** | | **ADAS-Cog13**  ***r*^2^ = 0.50** | | **RBANS**  ***r*^2^ = 0.26** | |
| --- | --- | --- | --- | --- | --- | --- | --- | --- |
| **Covariate (units)** | **B (SE)** | ***p*** | **B (SE)** | ***p*** | **B (SE)** | ***p*** | **B (SE)** | ***p*** |
| Baseline score  (1 point) | 0.03 (0.07) | 0.634 | 0.16 (0.09) | 0.093 | 0.08 (0.06) | 0.199 | -0.13 (0.07) | 0.063 |
| Age  (10 years) | -0.49 (0.39) | 0.212 | 0.65 (0.25) | ***0.012*** | 0.51 (0.73) | 0.488 | -1.66 (1.14) | 0.151 |
| [^18^F]florbetapir  (cortex; 0.1 SUVR) | -0.05 (0.19) | 0.781 | -0.11 (0.13) | 0.396 | 0.03 (0.36) | 0.937 | 0.81 (0.56) | 0.150 |
| MRI (cortical volume; cm^3^) | 0.01 (0.01) | 0.495 | -0.01 (0.01) | 0.136 | -0.03 (0.01) | 0.087 | -0.02 (0.02) | 0.379 |
| [^18^F]GTP1  (WCG; 0.1 SUVR) | -0.21 (0.13) | 0.118 | 0.11 (0.08) | 0.191 | 0.36 (0.25) | 0.153 | -1.58 (0.47) | ***0.001*** |

| **Braak I/II ROI** | **MMSE**  ***r*^2^ = 0.34** | | **CDR-SB**  ***r*^2^ = 0.33** | | **ADAS-Cog13**  ***r*^2^ = 0.49** | | **RBANS**  ***r*^2^ = 0.23** | |
| --- | --- | --- | --- | --- | --- | --- | --- | --- |
| **Covariate (units)** | **B (SE)** | ***p*** | **B (SE)** | ***p*** | **B (SE)** | ***p*** | **B (SE)** | ***p*** |
| Baseline score  (1 point) | 0.09 (0.07) | 0.192 | 0.27 (0.09) | ***0.003*** | 0.14 (0.05) | ***0.012*** | -0.05 (0.06) | 0.379 |
| Age  (10 years) | -0.53 (0.40) | 0.189 | 0.62 (0.25) | ***0.017*** | 0.48 (0.74) | 0.522 | -1.74 (1.17) | 0.143 |
| [^18^F]florbetapir  (cortex; 0.1 SUVR) | -0.14 (0.20) | 0.465 | 0.02 (0.13) | 0.883 | 0.27 (0.36) | 0.451 | 0.79 (0.58) | 0.175 |
| MRI (cortical volume; cm^3^) | 0.01 (0.01) | 0.205 | -0.01 (0.00) | ***0.017*** | -0.03 (0.01) | ***0.022*** | -0.00 (0.02) | 0.934 |
| [^18^F]GTP1  (WCG; 0.1 SUVR) | -0.03 (0.13) | 0.811 | -0.13 (0.08) | 0.089 | -0.14 (0.23) | 0.541 | -1.15 (0.41) | ***0.007*** |

| **Braak III/IV ROI** | **MMSE**  ***r*^2^ = 0.40** | | **CDR-SB**  ***r*^2^ = 0.35** | | **ADAS-Cog13**  ***r*^2^ = 0.52** | | **RBANS**  ***r*^2^ = 0.26** | |
| --- | --- | --- | --- | --- | --- | --- | --- | --- |
| **Covariate (units)** | **B (SE)** | ***p*** | **B (SE)** | ***p*** | **B (SE)** | ***p*** | **B (SE)** | ***p*** |
| Baseline score  (1 point) | 0.02 (0.06) | 0.835 | 0.13 (0.09) | 0.162 | 0.05 (0.05) | 0.326 | -0.13 (0.07) | 0.061 |
| Age  (10 years) | -0.75 (0.39) | 0.058 | 0.80 (0.26) | ***0.003*** | 0.89 (0.74) | 0.233 | -2.41 (1.15) | ***0.041*** |
| [^18^F]florbetapir  (cortex; 0.1 SUVR) | 0.04 (0.19) | 0.835 | -0.16 (0.13) | 0.205 | -0.14 (0.35) | 0.696 | 0.83 (0.56) | 0.139 |
| MRI (cortical volume; cm^3^) | -0.00 (0.01) | 0.949 | -0.00 (0.01) | 0.386 | -0.02 (0.01) | 0.245 | -0.03 (0.02) | 0.219 |
| [^18^F]GTP1  (WCG; 0.1 SUVR) | -0.39 (0.14) | ***0.006*** | 0.21 (0.09) | ***0.025*** | 0.69 (0.28) | ***0.015*** | -1.96 (0.58) | ***0.001*** |

| **Braak V/VI ROI** | **MMSE**  ***r*^2^ = 0.36** | | **CDR-SB**  ***r*^2^ = 0.33** | | **ADAS-Cog13**  ***r*^2^ = 0.51** | | **RBANS**  ***r*^2^ = 0.19** | |
| --- | --- | --- | --- | --- | --- | --- | --- | --- |
| **Covariate (units)** | **B (SE)** | ***p*** | **B (SE)** | ***p*** | **B (SE)** | ***p*** | **B (SE)** | ***p*** |
| Baseline score  (1 point) | 0.07 (0.06) | 0.243 | 0.16 (0.09) | 0.061 | 0.09 (0.05) | 0.077 | -0.04 (0.06) | 0.504 |
| Age  (10 years) | -0.83 (0.42) | 0.050 | 0.83 (0.28) | ***0.003*** | 1.08 (0.77) | 0.163 | -2.88 (1.24) | ***0.023*** |
| [^18^F]florbetapir  (cortex; 0.1 SUVR) | -0.06 (0.19) | 0.742 | -0.11 (0.12) | 0.367 | -0.02 (0.34) | 0.942 | 0.65 (0.56) | 0.254 |
| MRI (cortical volume; cm^3^) | 0.00 (0.01) | 0.781 | -0.01 (0.01) | 0.226 | -0.02 (0.01) | 0.224 | -0.02 (0.03) | 0.344 |
| [^18^F]GTP1  (WCG; 0.1 SUVR) | -0.24 (0.12) | ***0.046*** | 0.14 (0.08) | 0.079 | 0.56 (0.23) | ***0.016*** | -1.71 (0.66) | ***0.012*** |

Abbreviations: MMSE, Mini-Mental State Examination; CDR-SB, Clinical Dementia Rating Sum of Boxes; ADAS-Cog13, 13-item version of the Alzheimer’s Disease Assessment Scale-Cognitive Subscale; RBANS, Repeatable Battery for the Assessment of Neuropsychological Status; WCG, whole cortical gray.

**Supplemental Table 3:** Linear mixed effects models assessing change on cognitive indices from baseline incorporating demographic, baseline cognitive, and imaging variables (using temporal, Braak I/II, Braak III/IV, and Braak V/VI ROIs and all participants). Partial regression coefficients (B), standard errors (SE), and *p* values are reported for each predictor; *r*^2^ values are reported for each linear mixed effect model.

| **Temporal ROI** | **MMSE**  ***r*^2^ = 0.37** | | **CDR-SB**  ***r*^2^ = 0.36** | | **ADAS-Cog13**  ***r*^2^ = 0.54** | | **RBANS**  ***r*^2^ = 0.27** | |
| --- | --- | --- | --- | --- | --- | --- | --- | --- |
| **Covariate (units)** | **B (SE)** | ***p*** | **B (SE)** | ***p*** | **B (SE)** | ***p*** | **B (SE)** | ***p*** |
| Baseline score  (1 point) | 0.05 (0.06) | 0.478 | 0.20 (0.08) | ***0.011*** | 0.06 (0.05) | 0.253 | -0.07 (0.07) | 0.308 |
| Age  (10 years) | -0.39 (0.34) | 0.252 | 0.60 (0.22) | ***0.006*** | 0.86 (0.66) | 0.199 | -1.40 (1.13) | 0.221 |
| [^18^F]florbetapir  (cortex; 0.1 SUVR) | -0.07 (0.16) | 0.660 | -0.08 (0.10) | 0.45 | 0.10 (0.30) | 0.743 | 0.23 (0.51) | 0.660 |
| MRI (cortical volume; cm^3^) | 0.00 (0.01) | 0.702 | -0.01 (0.00) | 0.213 | -0.03 (0.01) | ***0.038*** | -0.04 (0.02) | 0.076 |
| [^18^F]GTP1  (WCG; 0.1 SUVR) | -0.22 (0.12) | 0.073 | 0.11 (0.07) | 0.138 | 0.42 (0.24) | 0.081 | -1.48 (0.46) | ***0.002*** |

| **Braak I/II ROI** | **MMSE**  ***r*^2^ = 0.35** | | **CDR-SB**  ***r*^2^ = 0.36** | | **ADAS-Cog13**  ***r*^2^ = 0.52** | | **RBANS**  ***r*^2^ = 0.23** | |
| --- | --- | --- | --- | --- | --- | --- | --- | --- |
| **Covariate (units)** | **B (SE)** | ***p*** | **B (SE)** | ***p*** | **B (SE)** | ***p*** | **B (SE)** | ***p*** |
| Baseline score  (1 point) | 0.11 (0.06) | 0.064 | 0.31 (0.07) | ***<0.001*** | 0.12 (0.05) | ***0.010*** | 0.00 (0.06) | 0.973 |
| Age  (10 years) | -0.44 (0.35) | 0.210 | 0.56 (0.22) | ***0.011*** | 0.86 (0.67) | 0.207 | -1.64 (1.16) | 0.160 |
| [^18^F]florbetapir  (cortex; 0.1 SUVR) | -0.18 (0.16) | 0.252 | 0.05 (0.10) | 0.648 | 0.34 (0.30) | 0.264 | 0.10 (0.52) | 0.856 |
| MRI (cortical volume; cm^3^) | 0.01 (0.01) | 0.328 | -0.01 (0.00) | ***0.016*** | -0.03 (0.01) | ***0.005*** | -0.03 (0.02) | 0.160 |
| [^18^F]GTP1  (WCG; 0.1 SUVR) | -0.01 (0.11) | 0.904 | -0.13 (0.07) | 0.053 | -0.09 (0.21) | 0.679 | -1.05 (0.42) | ***0.015*** |

| **Braak III/IV ROI** | **MMSE**  ***r*^2^ = 0.41** | | **CDR-SB**  ***r*^2^ = 0.38** | | **ADAS-Cog13**  ***r*^2^ = 0.55** | | **RBANS**  ***r*^2^ = 0.25** | |
| --- | --- | --- | --- | --- | --- | --- | --- | --- |
| **Covariate (units)** | **B (SE)** | ***p*** | **B (SE)** | ***p*** | **B (SE)** | ***p*** | **B (SE)** | ***p*** |
| Baseline score  (1 point) | 0.03 (0.06) | 0.601 | 0.17 (0.08) | ***0.026*** | 0.04 (0.05) | 0.388 | -0.05 (0.07) | 0.405 |
| Age  (10 years) | -0.60 (0.34) | 0.077 | 0.73 (0.22) | ***0.001*** | 1.18 (0.66) | 0.078 | -1.89 (1.14) | 0.101 |
| [^18^F]florbetapir  (cortex; 0.1 SUVR) | 0.02 (0.16) | 0.888 | -0.12 (0.10) | 0.226 | -0.05 (0.30) | 0.872 | 0.21 (0.52) | 0.683 |
| MRI (cortical volume; cm^3^) | -0.00 (0.01) | 0.811 | -0.00 (0.00) | 0.482 | -0.02 (0.01) | 0.105 | -0.04 (0.02) | 0.067 |
| [^18^F]GTP1  (WCG; 0.1 SUVR) | -0.38 (0.12) | ***0.003*** | 0.20 (0.08) | ***0.015*** | 0.71 (0.26) | ***0.007*** | -1.66 (0.55) | ***0.004*** |

| **Braak V/VI ROI** | **MMSE**  ***r*^2^ = 0.37** | | **CDR-SB**  ***r*^2^ = 0.36** | | **ADAS-Cog13**  ***r*^2^ = 0.54** | | **RBANS**  ***r*^2^ = 0.19** | |
| --- | --- | --- | --- | --- | --- | --- | --- | --- |
| **Covariate (units)** | **B (SE)** | ***p*** | **B (SE)** | ***p*** | **B (SE)** | ***p*** | **B (SE)** | ***p*** |
| Baseline score  (1 point) | 0.08 (0.05) | 0.118 | 0.21 (0.07) | ***0.003*** | 0.08 (0.04) | 0.075 | 0.02 (0.06) | 0.663 |
| Age  (10 years) | -0.67 (0.36) | 0.065 | 0.75 (0.23) | ***0.002*** | 1.35 (0.68) | 0.051 | -2.15 (1.19) | 0.07 |
| [^18^F]florbetapir  (cortex; 0.1 SUVR) | -0.09 (0.15) | 0.547 | -0.07 (0.10) | 0.478 | 0.08 (0.29) | 0.772 | -0.02 (0.52) | 0.971 |
| MRI (cortical volume; cm^3^) | 0.00 (0.01) | 0.896 | -0.00 (0.00) | 0.278 | -0.02 (0.01) | 0.083 | -0.04 (0.02) | 0.112 |
| [^18^F]GTP1  (WCG; 0.1 SUVR) | -0.22 (0.10) | ***0.038*** | 0.13 (0.07) | 0.070 | 0.56 (0.21) | ***0.010*** | -1.32 (0.63) | ***0.041*** |

Abbreviations: MMSE, Mini-Mental State Examination; CDR-SB, Clinical Dementia Rating Sum of Boxes; ADAS-Cog13, 13-item version of the Alzheimer’s Disease Assessment Scale-Cognitive Subscale; RBANS, Repeatable Battery for the Assessment of Neuropsychological Status; WCG, whole cortical gray.

**Supplemental Table 4.** Sensitivity and specificity of [^18^F]GTP1 standardized uptake value ratio (SUVR) cut points for distinguishing between decliners and non-decliners.

|  | **MMSE** | | **CDR-SB** | | **ADAS-Cog13** | |
| --- | --- | --- | --- | --- | --- | --- |
|  | **Sensitivity** | **Specificity** | **Sensitivity** | **Specificity** | **Sensitivity** | **Specificity** |
| **Distribution-based Cut Points** | | | | | | |
| WCG ROI (SUVR≥1.245) | 0.67 | 0.60 | 0.74 | 0.82 | 0.71 | 0.69 |
| Temporal ROI (SUVR≥1.325) | 0.89 | 0.43 | 0.91 | 0.59 | 0.90 | 0.50 |
| Braak I/II ROI  (SUVR≥1.586) | 0.48 | 0.77 | 0.43 | 0.77 | 0.48 | 0.81 |
| Braak III/IV ROI  (SUVR≥1.268) | 0.78 | 0.53 | 0.86 | 0.77 | 0.81 | 0.62 |
| Braak V/VI ROI  (SUVR≥1.232) | 0.52 | 0.70 | 0.60 | 0.91 | 0.61 | 0.85 |
| **Empirically determined Cut Points** | | | | | | |
| WCG ROI |  |  |  |  |  |  |
| MMSE  (SUVR≥1.194) | 0.81 | 0.53 | -- | -- | -- | -- |
| CDR-SB  (SUVR≥1.286) | -- | -- | 0.69 | 0.91 | -- | -- |
| ADAS-Cog13  (SUVR≥1.286) | -- | -- | -- | -- | 0.71 | 0.84 |
| Temporal ROI |  |  |  |  |  |  |
| MMSE  (SUVR≥1.533) | 0.67 | 0.77 | -- | -- | -- | -- |
| CDR-SB  (SUVR≥1.406) | -- | -- | 0.80 | 0.82 | -- | -- |
| ADAS-Cog13  (SUVR≥1.526) | -- | -- | -- | -- | 0.71 | 0.85 |
| Braak I/II ROI |  |  |  |  |  |  |
| MMSE  (SUVR≥1.562) | 0.77 | 0.56 | -- | -- | -- | -- |
| CDR-SB  (SUVR≥1.497) | -- | -- | 0.77 | 0.63 | -- | -- |
| ADAS-Cog13  (SUVR≥1. 497) | -- | -- | -- | -- | 0.81 | 0.71 |
| Braak III/IV ROI |  |  |  |  |  |  |
| MMSE  (SUVR≥1.412) | 0.70 | 0.74 | -- | -- | -- | -- |
| CDR-SB  (SUVR≥1.269) | -- | -- | 0.77 | 0.86 | -- | -- |
| ADAS-Cog13  (SUVR≥1.333) | -- | -- | -- | -- | 0.69 | 0.77 |
| Braak V/VI ROI |  |  |  |  |  |  |
| MMSE  (SUVR≥1.184) | 0.60 | 0.74 | -- | -- | -- | -- |
| CDR-SB  (SUVR≥1.232) | -- | -- | 0.91 | 0.60 | -- | -- |
| ADAS-Cog13  (SUVR≥1.248) | -- | -- | -- | -- | 0.88 | 0.58 |

Abbreviations: MMSE, Mini-Mental State Examination; CDR-SB, Clinical Dementia Rating Sum of Boxes; ADAS-Cog13, 13-item version of the Alzheimer’s Disease Assessment Scale-Cognitive Subscale; ROI, region of interest; WCG, whole cortical gray.

**Supplementary Table 5:** Odds ratios (ORs) for participants with prodromal, mild, or moderate AD experiencing cognitive decline that meets Minimal Clinically Important Differences (MCIDs) for participants with [^18^F]GTP1 SUVRs above specified cut points in specified ROIs.

|  | **MMSE** | | **CDR-SB** | | **ADAS-Cog13** | |
| --- | --- | --- | --- | --- | --- | --- |
|  | **OR** | ***p*** | **OR** | ***p*** | **OR** | ***p*** |
| **Distribution-based Cut Points** | | | | | | |
| WCG ROI (SUVR≥1.245) | 3.00 | ***0.047*** | 13.00 | ***<0.001*** | 5.50 | ***0.003*** |
| Temporal ROI (SUVR≥1.325) | 6.18 | ***0.011*** | 15.41 | ***<0.001*** | 9.33 | ***0.002*** |
| Braak I/II ROI  (SUVR≥1.586) | 3.05 | 0.054 | 2.55 | 0.127 | 3.94 | ***0.026*** |
| Braak III/IV ROI  (SUVR≥1.268) | 4.00 | ***0.019*** | 20.40 | ***<0.001*** | 6.67 | ***0.002*** |
| Braak V/VI ROI  (SUVR≥1.232) | 2.51 | 0.096 | 15.00 | ***0.001*** | 8.71 | ***0.001*** |
| **Empirically determined Cut Points** | | | | | | |
| WCG ROI |  |  |  |  |  |  |
| MMSE  (SUVR≥1.194) | 5.03 | ***0.009*** | -- | -- | -- | -- |
| CDR-SB  (SUVR≥1.286) | -- | -- | 21.82 | ***<0.001*** | -- | -- |
| ADAS-Cog13  (SUVR≥1.286) | -- | -- | -- | -- | 13.44 | ***<0.001*** |
| Temporal ROI |  |  |  |  |  |  |
| MMSE  (SUVR≥1.533) | 6.57 | ***0.002*** | -- | -- | -- | -- |
| CDR-SB  (SUVR≥1.406) | -- | -- | 18.00 | ***<0.001*** | -- | -- |
| ADAS-Cog13  (SUVR≥1.526) | -- | -- | -- | -- | 13.44 | ***<0.001*** |
| Braak I/II ROI |  |  |  |  |  |  |
| MMSE  (SUVR≥1.562) | 4.11 | ***0.015*** | -- | -- | -- | -- |
| CDR-SB  (SUVR≥1.497) | -- | -- | 7.75 | ***0.005*** | -- | -- |
| ADAS-Cog13  (SUVR≥1. 497) | -- | -- | -- | -- | 10.27 | ***<0.001*** |
| Braak III/IV ROI |  |  |  |  |  |  |
| MMSE  (SUVR≥1.412) | 6.67 | ***0.001*** | -- | -- | -- | -- |
| CDR-SB  (SUVR≥1.269) | -- | -- | 20.40 | ***<0.001*** | -- | -- |
| ADAS-Cog13  (SUVR≥1.333) | -- | -- | -- | -- | 7.71 | ***0.001*** |
| Braak V/VI ROI |  |  |  |  |  |  |
| MMSE  (SUVR≥1.184) | 4.29 | ***0.012*** | -- | -- | -- | -- |
| CDR-SB  (SUVR≥1.232) | -- | -- | 15.00 | ***0.001*** | -- | -- |
| ADAS-Cog13  (SUVR≥1.248) | -- | -- | -- | -- | 10.62 | 0.001 |

Abbreviations: MMSE, Mini-Mental State Examination; CDR-SB, Clinical Dementia Rating Sum of Boxes; ADAS-Cog13, 13-item version of the Alzheimer’s Disease Assessment Scale-Cognitive Subscale; ROI, region of interest; WCG, whole cortical gray.
