# Supplementary figures and images for: Baseline [18F]GTP1 tau PET imaging is associated with subsequent cognitive decline in Alzheimer’s disease
Source: Alzheimers Res Ther. 2021 Dec 1;13:196. doi: 10.1186/s13195-021-00937-x (PMC8638526; doi:10.1186/s13195-021-00937-x)

MMSE

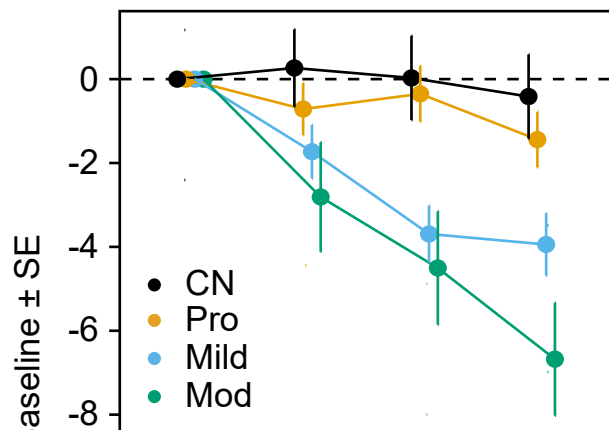

CDR-SB

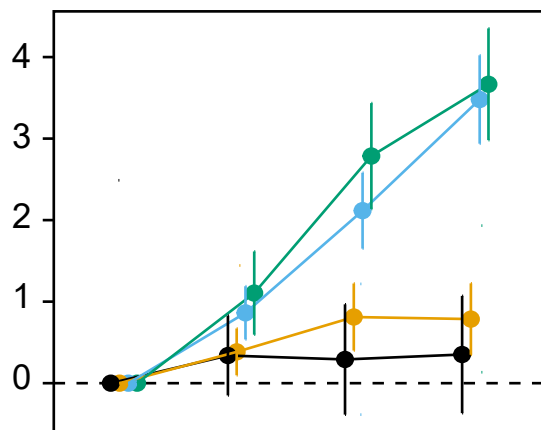

ADAS-Cog13

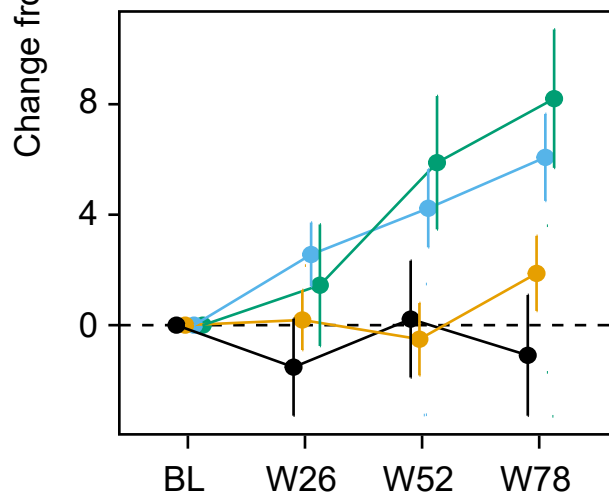

RBANS Total Index

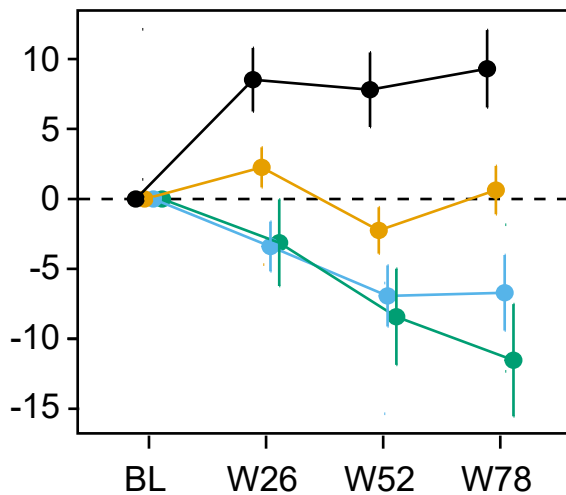

Visit

Supplement: Supplementary file 2 — Additional file 2: Supplemental Figure 1. Longitudinal change from baseline at each assessment timepoint using a mixed model repeated measure analysis on the Mini-Mental State Exam (MMSE), Clinical Dementia Rating Sum of Boxes (CDR-SB), 13-item version of the Alzheimer’s Disease Assessment Scale-Cognitive Subscale (ADAS-Cog13), and Repeatable Battery for the Assessment of Neuropsychological Status (RBANS) Total Index. SE: standard error; BL: baseline; W26: Week 26 visit; W52: Week 52 visit; W78: Week 78 visit. [file 13195_2021_937_MOESM2_ESM.pdf]

A.

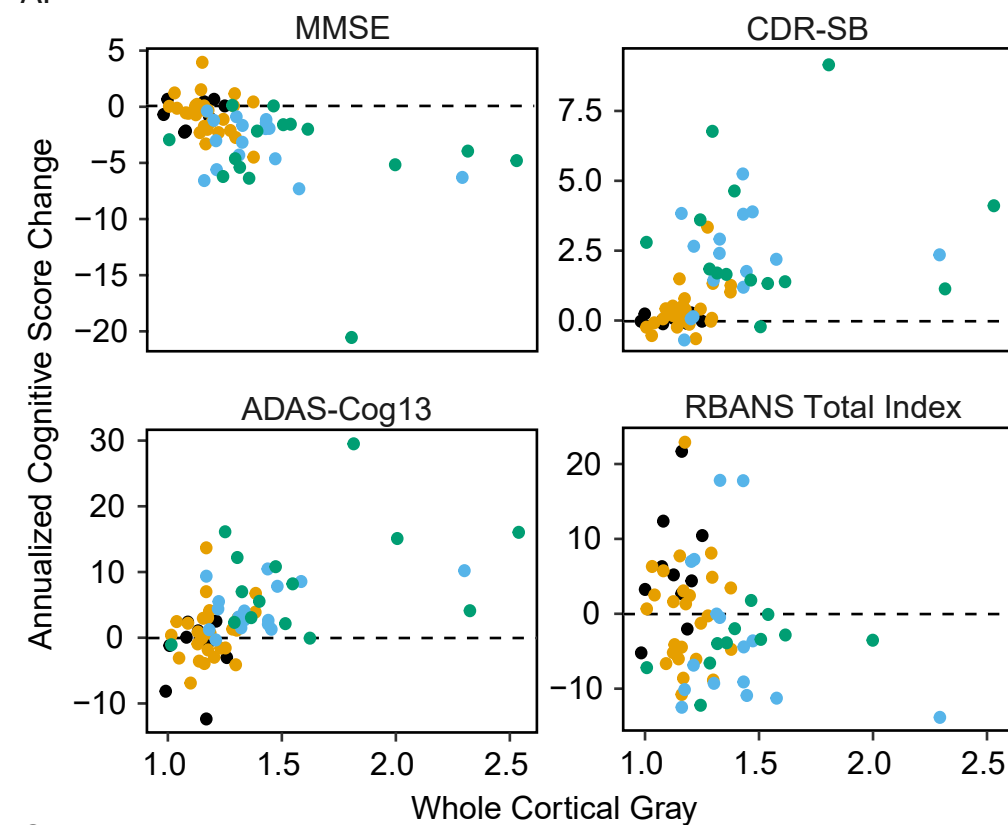

B.

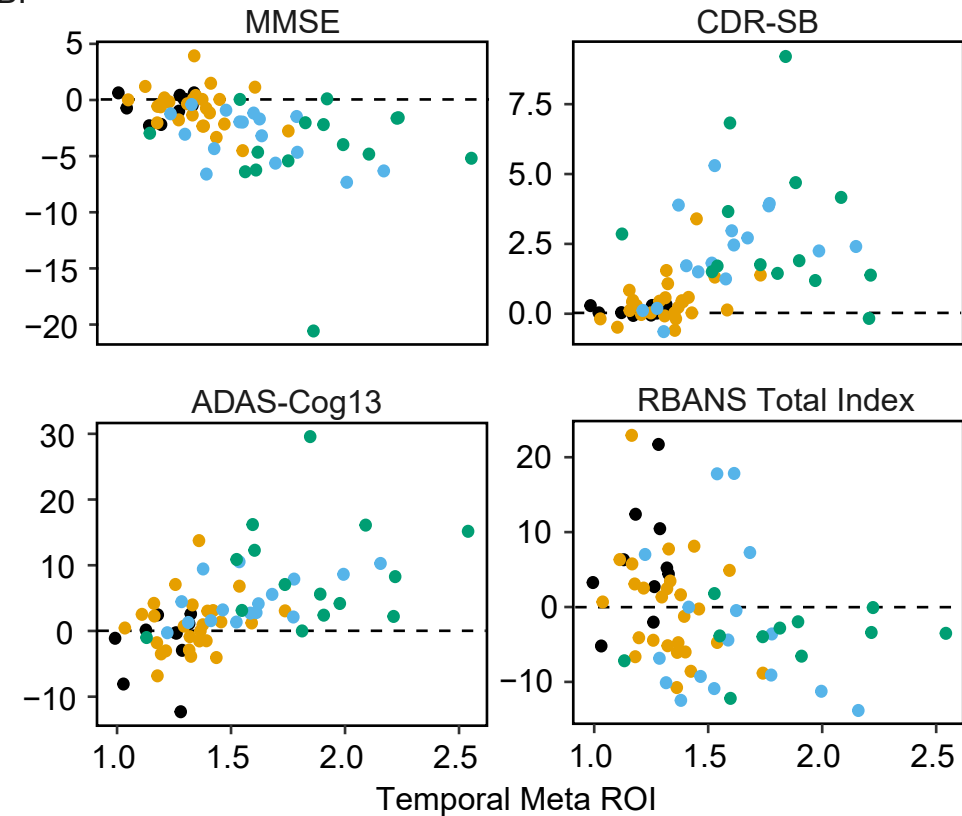

C.

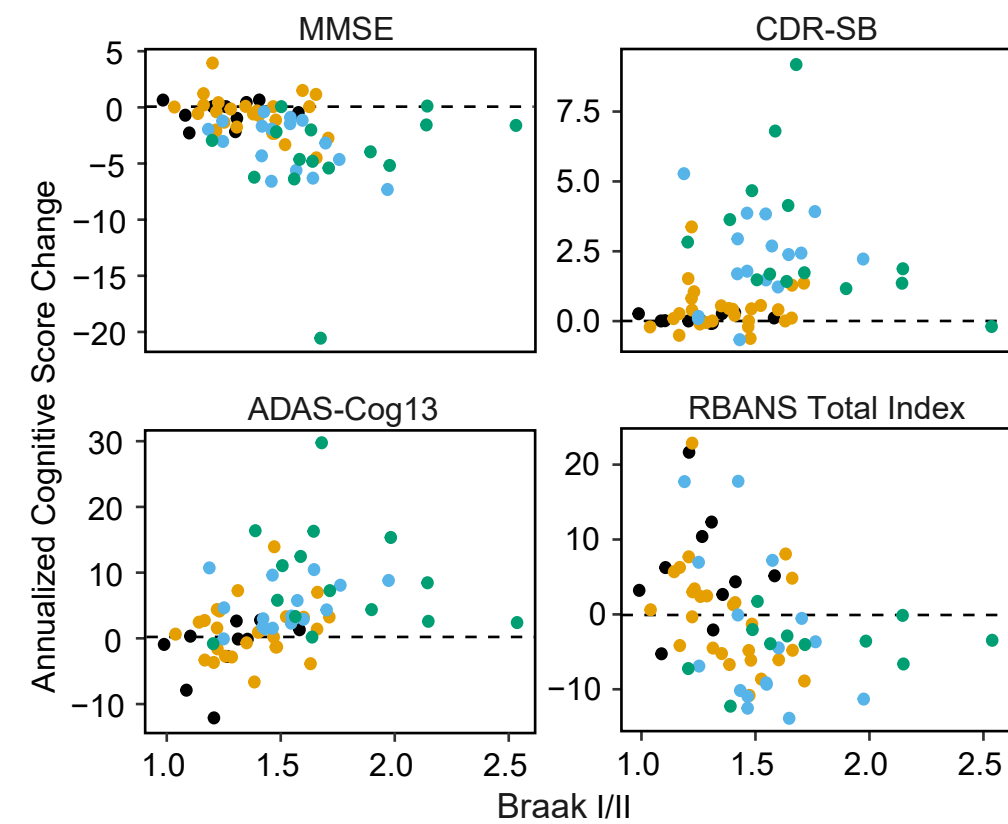

D.

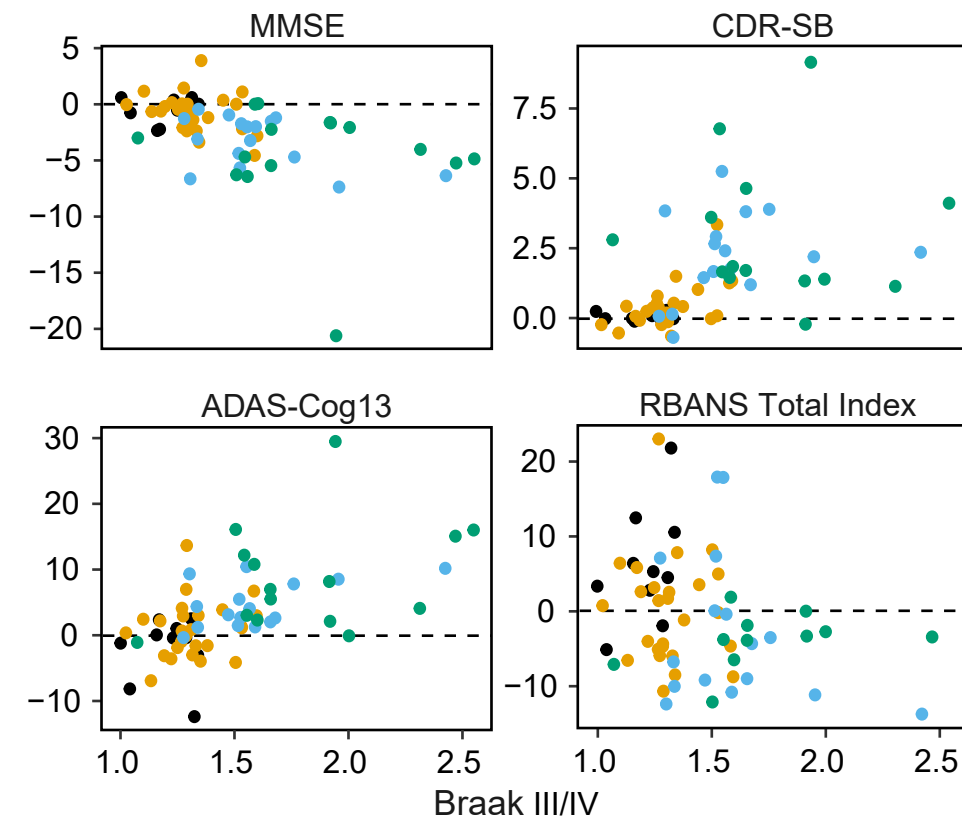

E.

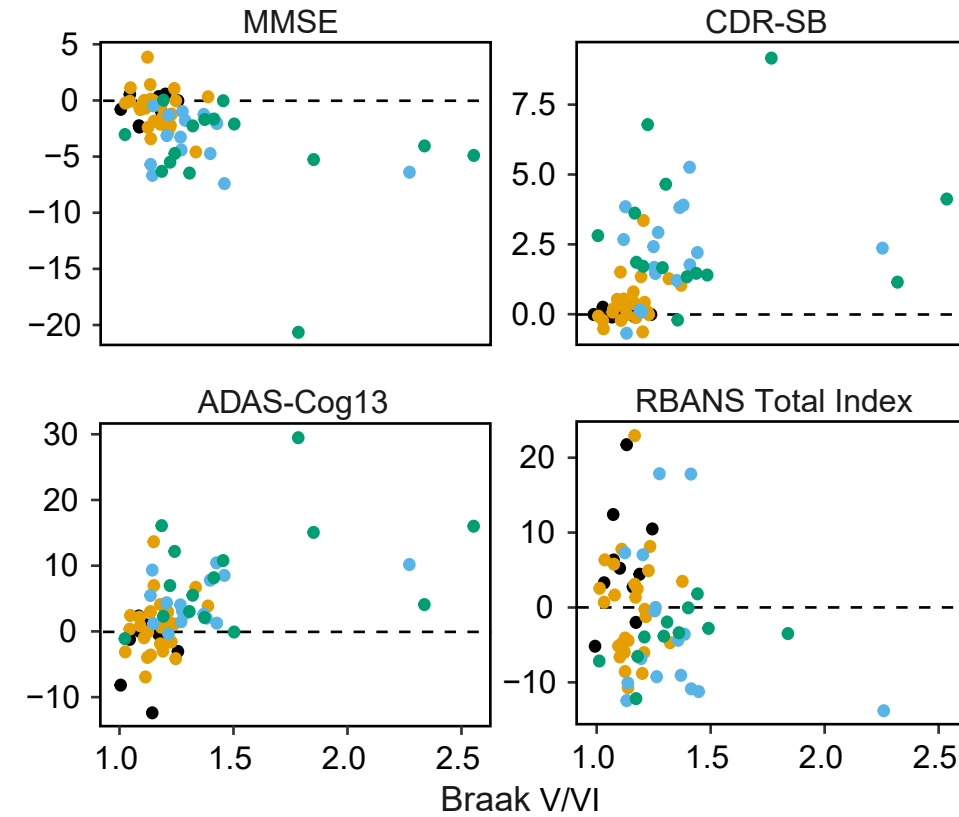

● CN  
● Pro  
● Mild  
● Mod

Supplement: Supplementary file 3 — Additional file 3: Supplemental Figure 2. Scatterplots of baseline [18F]GTP1 SUVR versus annualized change scores calculated via estimated slopes on the Mini-Mental State Exam (MMSE), Clinical Dementia Rating Sum of Boxes (CDR-SB), 13-item version of the Alzheimer’s Disease Assessment Scale-Cognitive Subscale (ADAS-Cog13), and Repeatable Battery for the Assessment of Neuropsychological Status (RBANS) Total Index in the cognitively normal (CN) and prodromal (Pro), mild (Mild), and moderate (Mod) AD groups in the (A) whole cortical gray, (B) temporal, (C) Braak I/II, (D) Braak III/IV, and (E) Braak V/VI ROIs. [file 13195_2021_937_MOESM3_ESM.pdf]

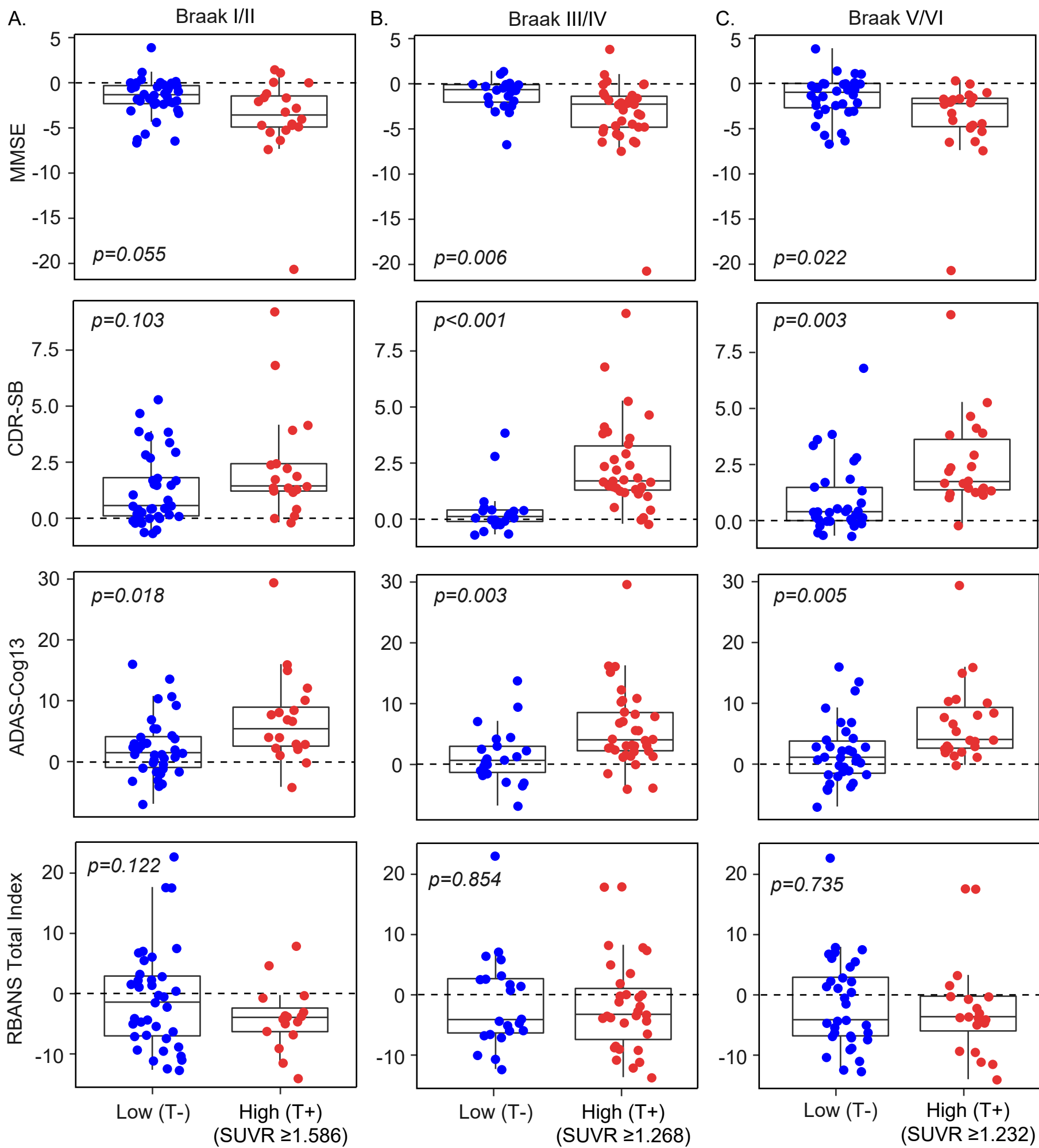

Supplement: Supplementary file 4 — Additional file 4: Supplemental Figure 3. Boxplots of annualized rates of change on the Mini-Mental State Exam (MMSE), Clinical Dementia Rating Sum of Boxes (CDR-SB), 13-item version of the Alzheimer’s Disease Assessment Scale-Cognitive Subscale (ADAS-Cog13), and Repeatable Battery for the Assessment of Neuropsychological Status (RBANS) Total Index for study participants dichotomized by distribution-based [18F]GTP1 SUVR cutoffs of (A) 1.586 in the Braak I/II ROI, (B) 1.268 in the Braak III/IV ROI, or (C) 1.232 in the Braak V/VI ROI. [file 13195_2021_937_MOESM4_ESM.pdf]
