# Supplementary material for: Baseline [18F]GTP1 tau PET imaging is associated with subsequent cognitive decline in Alzheimer’s disease
Source: Alzheimers Res Ther. 2021 Dec 1;13:196. doi: 10.1186/s13195-021-00937-x (PMC8638526; doi:10.1186/s13195-021-00937-x)
Supplement: Supplementary file 5 — Additional file 5: Supplemental Figure 4. Receiver Operating Characteristic curves for (A) Braak I/II, (B) Braak III/IV, and (C) Braak V/VI ROI [18F]GTP1 SUVRs for distinguishing between progressors and non-progressors on the Mini-Mental State Exam (MMSE), Clinical Dementia Rating Sum of Boxes (CDR-SB), and 13-item version of the Alzheimer’s Disease Assessment Scale-Cognitive Subscale (ADAS-Cog13). [file 13195_2021_937_MOESM5_ESM.pdf]

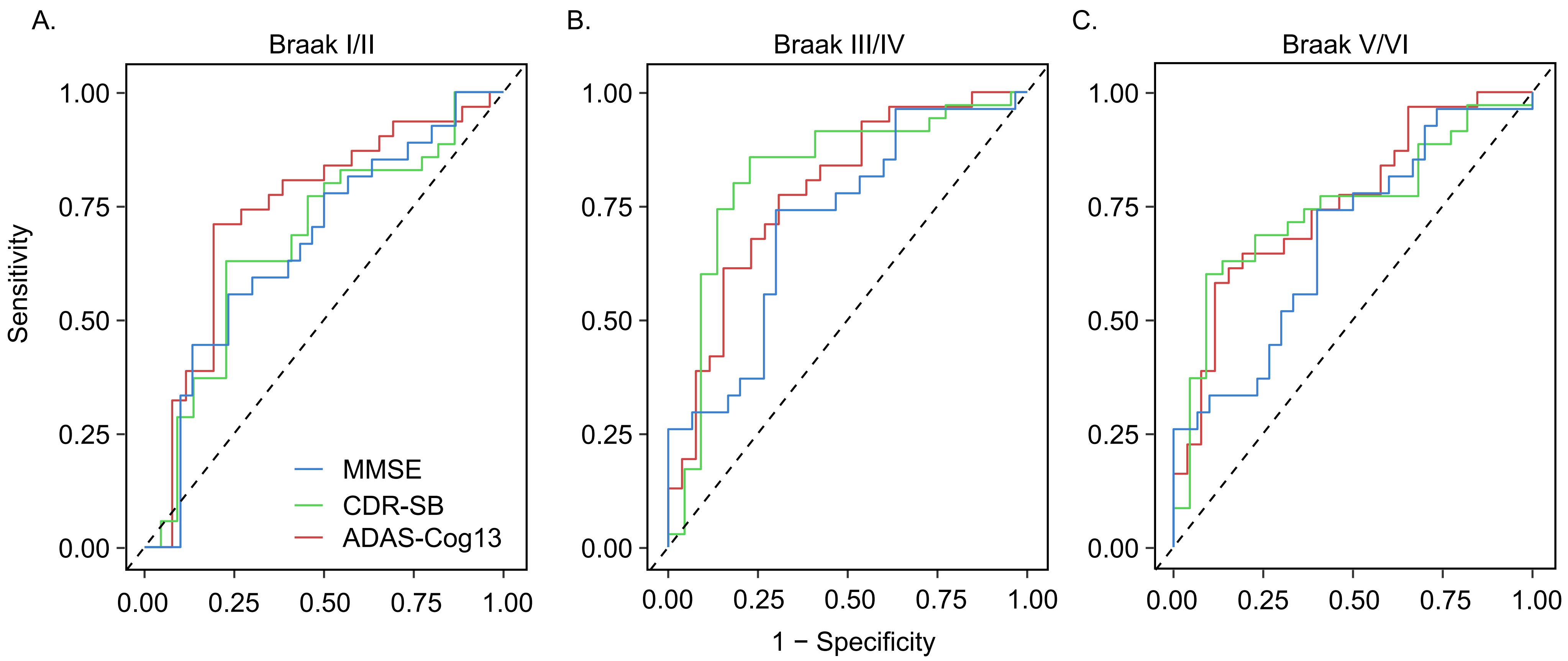

	Braak I/II		Braak III/IV		Braak V/VI	
	**AUC**	**95% CI**	**AUC**	**95%CI**	**AUC**	**95%CI**
MMSE	0.66	0.52-0.81	0.71	0.58-0.85	0.68	0.53-0.82
CDR-SB	0.67	0.52-0.82	0.82	0.69-0.95	0.74	0.61-0.87
ADAS-Cog13	0.74	0.60-0.87	0.78	0.66-0.90	0.75	0.53-0.82
